# Supplementary material for: Interplay of YEATS2 and GCDH regulates histone crotonylation and drives EMT in head and neck cancer
Source: eLife. 2025 Aug 14;14:RP103321. doi: 10.7554/eLife.103321 (PMC12352869; doi:10.7554/eLife.103321)
Supplement: Figure 3—source data 1. [file elife-103321-fig3-data1.zip › Figure 3—Source Data 1/Figure 3C and 3G.pdf]

Figure 3C

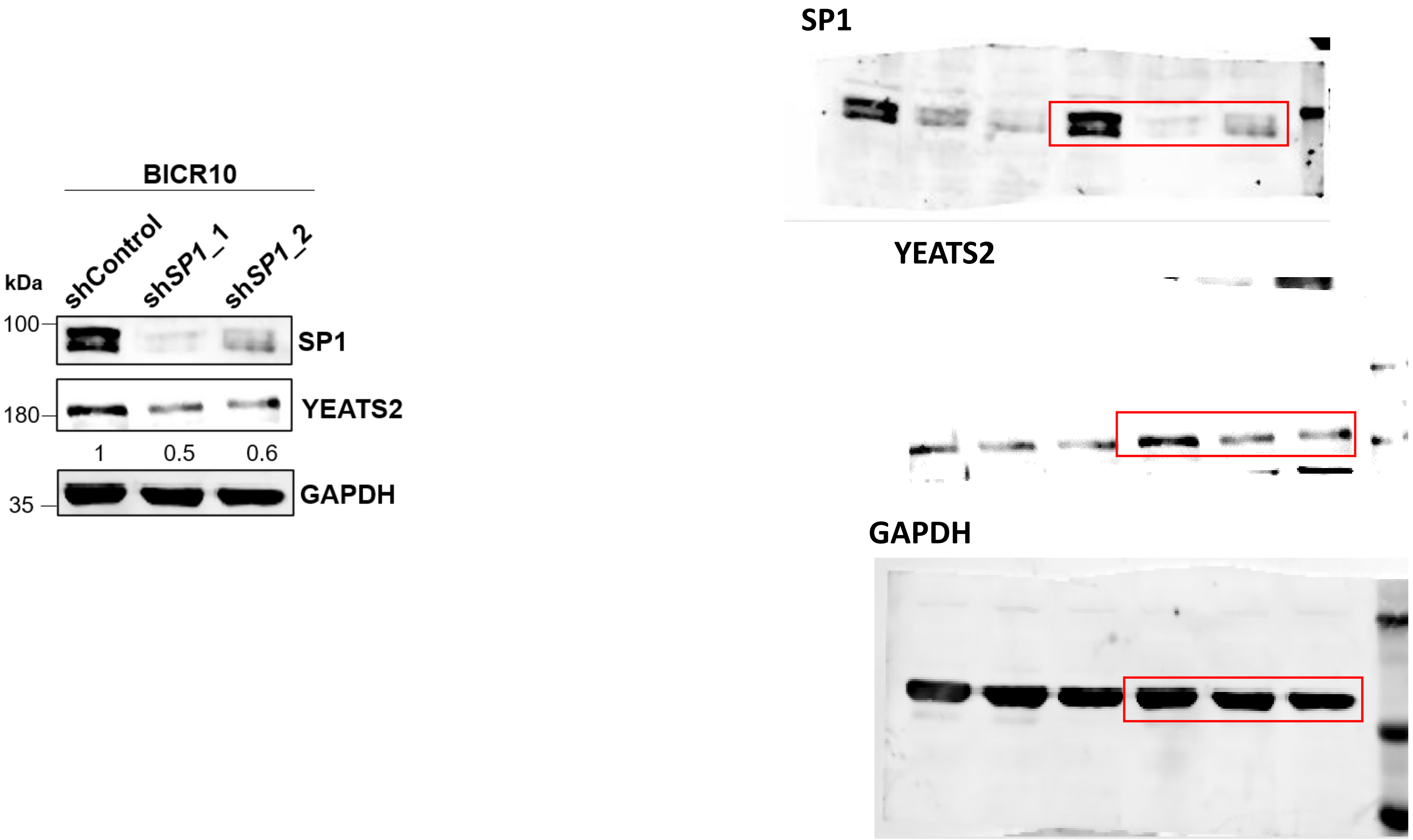

BICR10
kDa
shControl
shSP1_1
shSP1_2
100
SP1
180
YEATS2
1
0.5
0.6
35
GAPDH
SP1
YEATS2
GAPDH

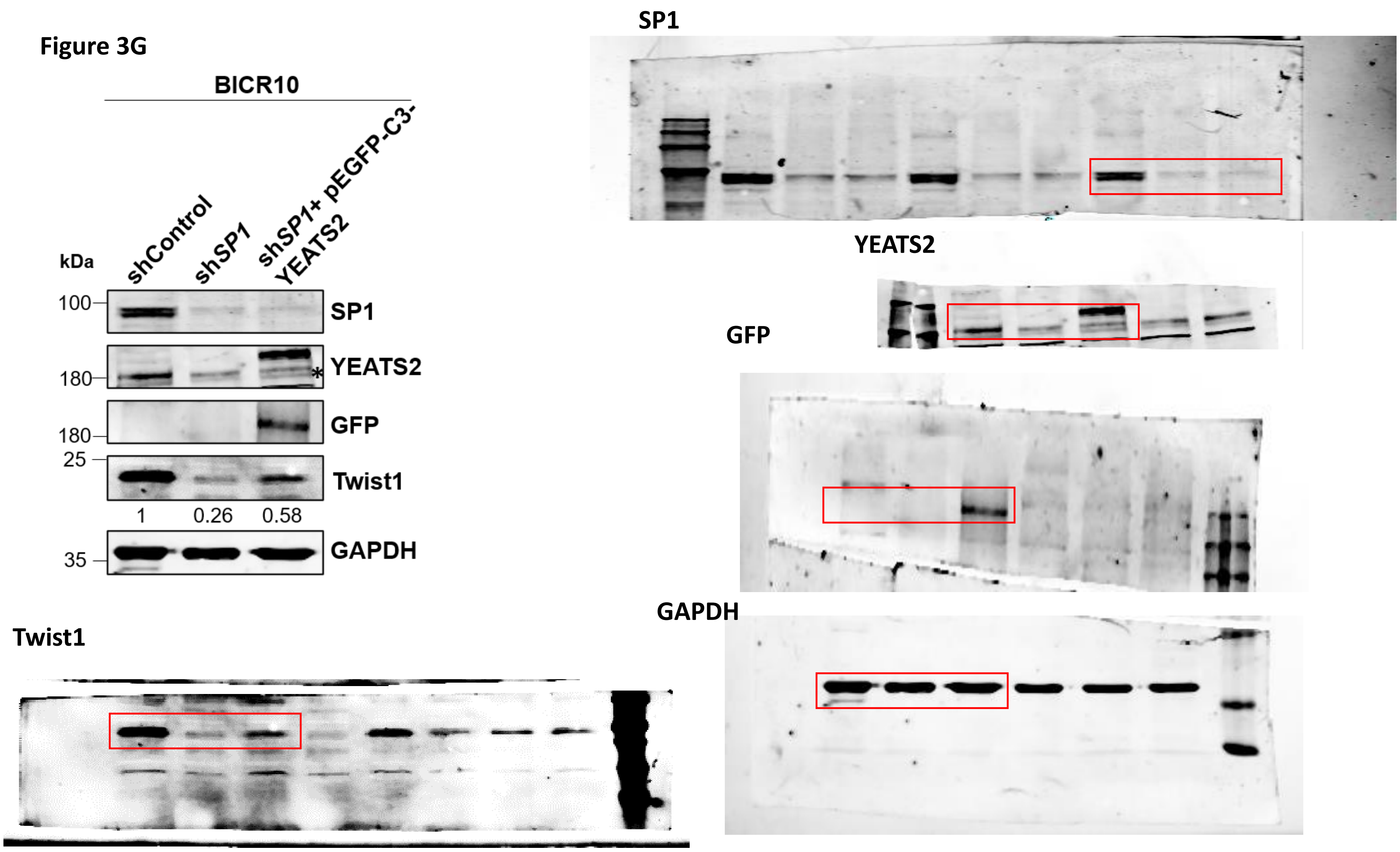

Figure 3—Source Data 1. PDF file containing original western blots for Figure 3C and 3G, indicating the relevant bands.
