# Supplementary material for: Interplay of YEATS2 and GCDH regulates histone crotonylation and drives EMT in head and neck cancer
Source: eLife. 2025 Aug 14;14:RP103321. doi: 10.7554/eLife.103321 (PMC12352869; doi:10.7554/eLife.103321)
Supplement: Figure 4—source data 1. [file elife-103321-fig4-data1.zip › Figure 4—Source Data 1/Figure 4D and 4G-H.pdf]

Figure 4D

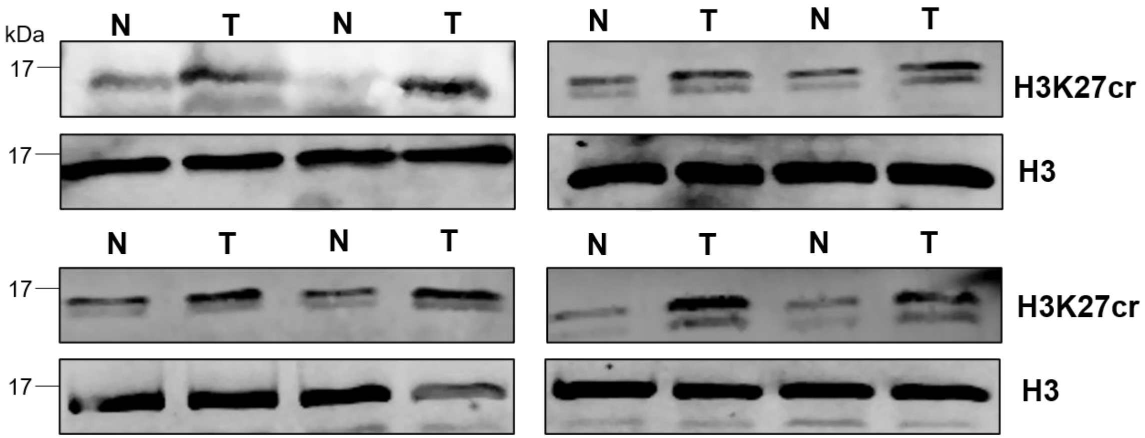
kDa
N T N T
N T N T
17
17
17
17
H3K27cr
H3
H3K27cr
H3

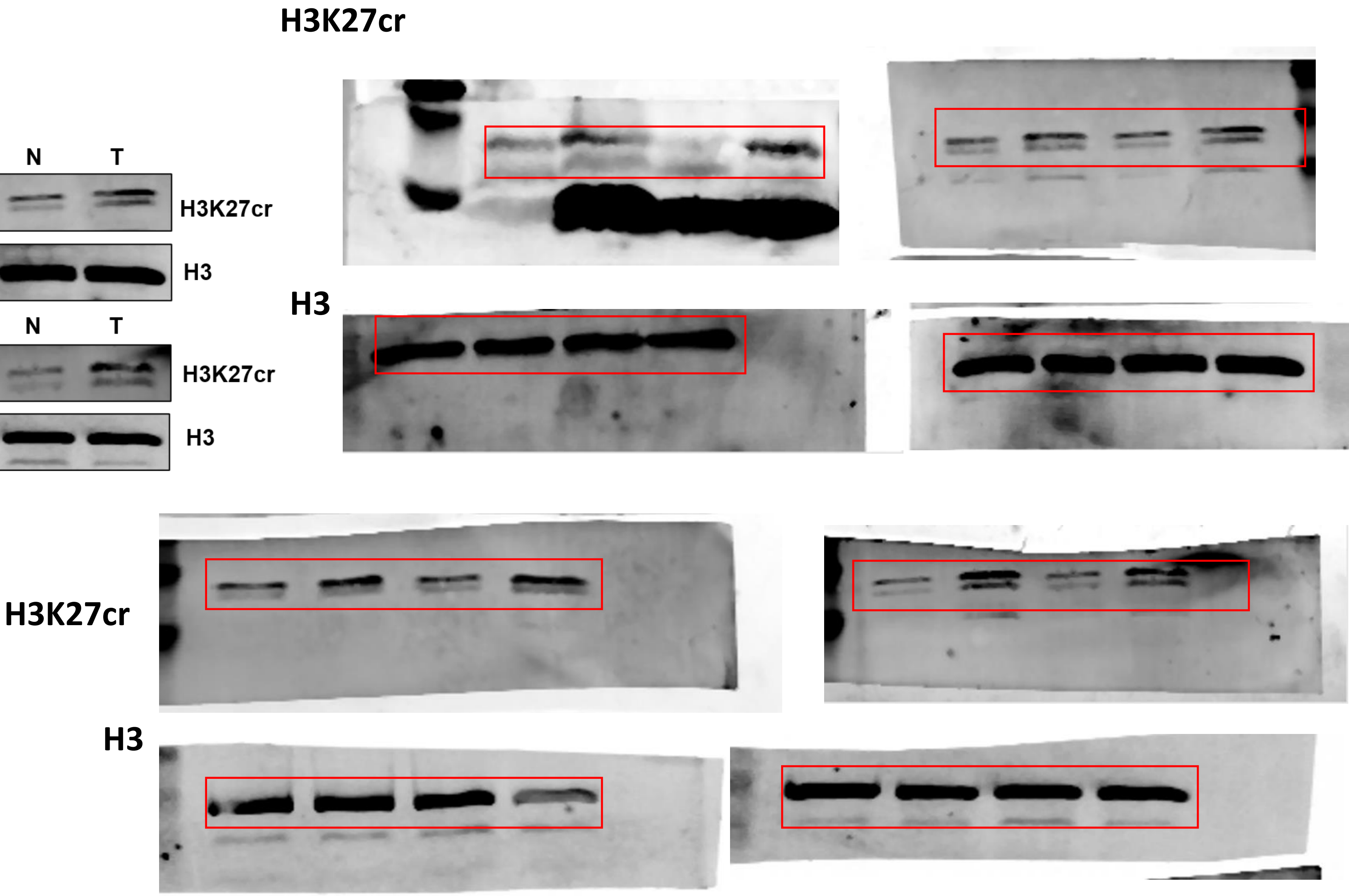
H3K27cr
H3
H3K27cr
H3

Figure 4G

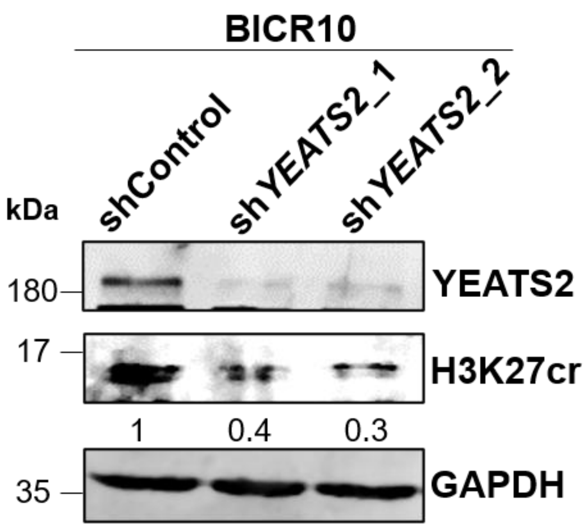

YEATS2

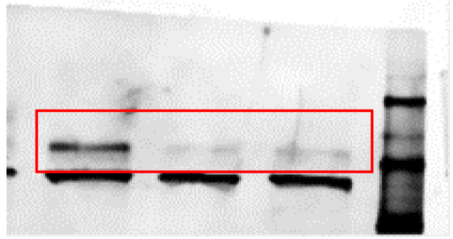

H3K27cr

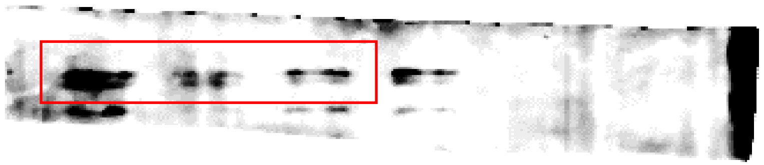

GAPDH

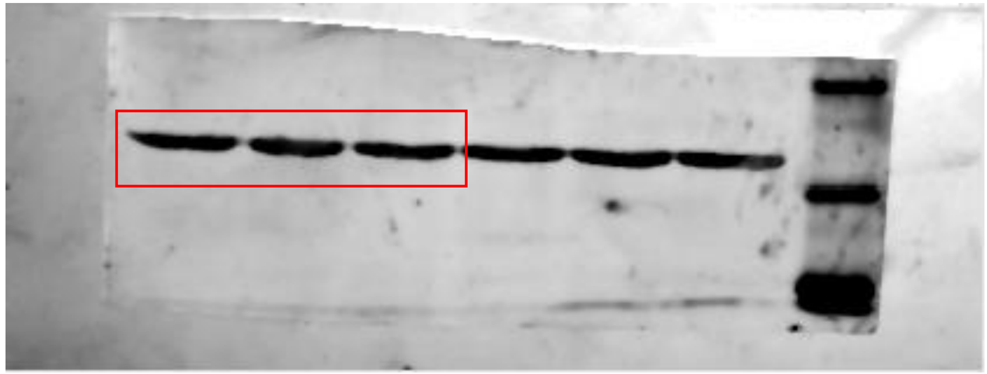

Figure 4H

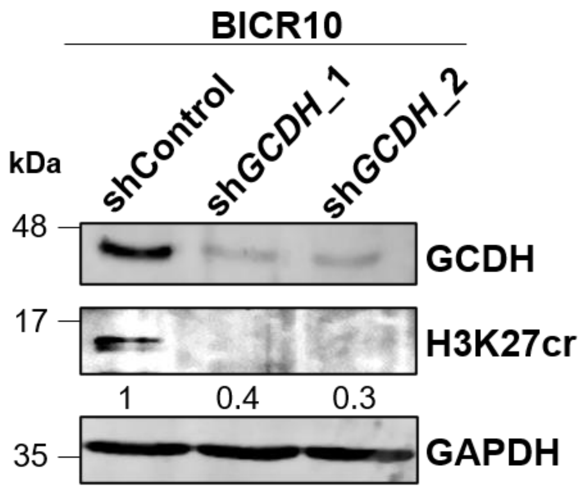

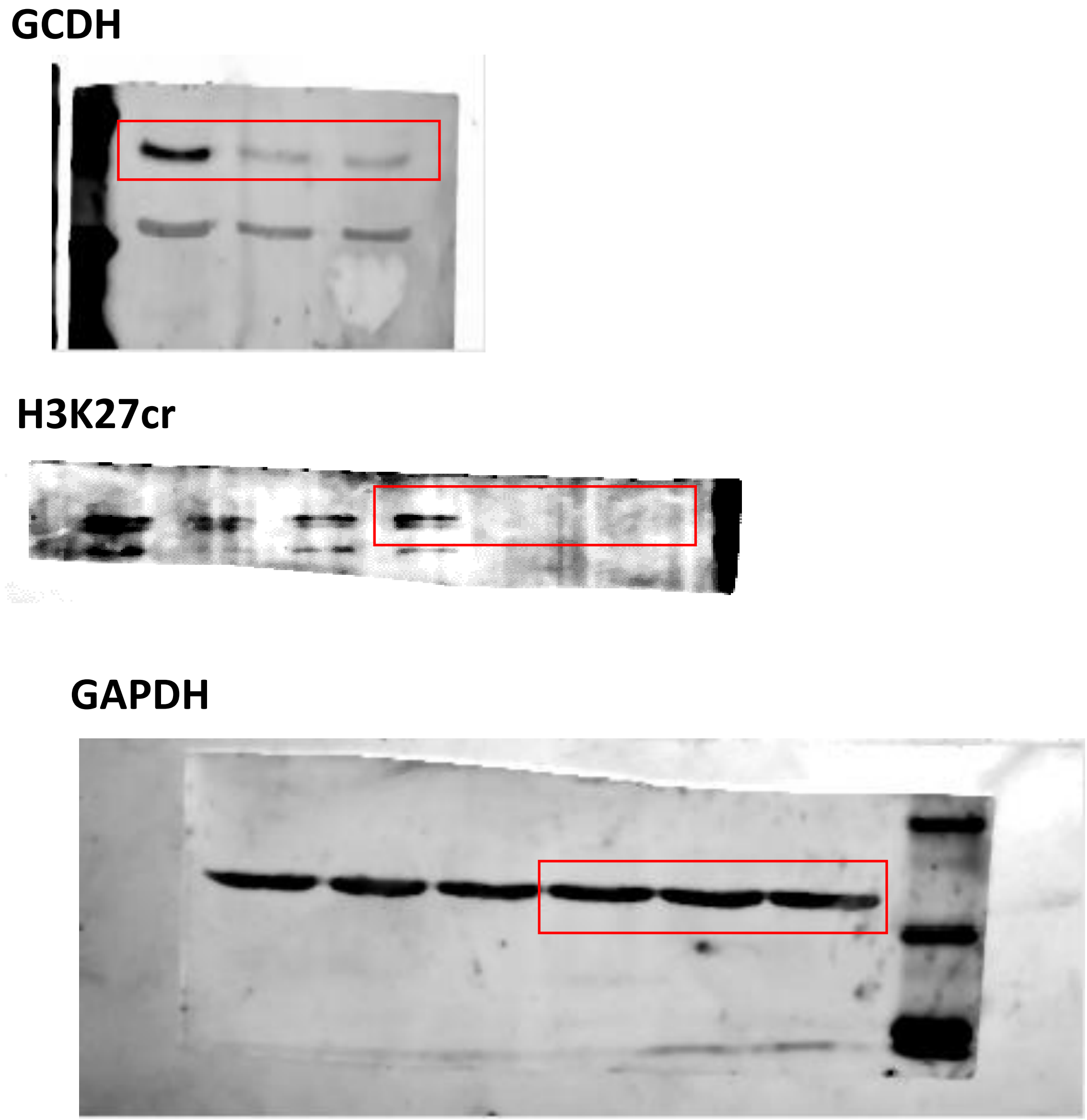

Figure 4—Source Data 1. PDF file containing original western blots for Figure 4D and 4G-H, indicating the relevant bands.
