# Supplementary material for: Interplay of YEATS2 and GCDH regulates histone crotonylation and drives EMT in head and neck cancer
Source: eLife. 2025 Aug 14;14:RP103321. doi: 10.7554/eLife.103321 (PMC12352869; doi:10.7554/eLife.103321)
Supplement: Figure 4—figure supplement 4—source data 1. [file elife-103321-fig4-figsupp4-data1.zip › Figure 4—figure supplement 4—Source Data 1/Figure 4-figure supplement 4A-B.pdf]

Figure 4- Figure Supplement 4A

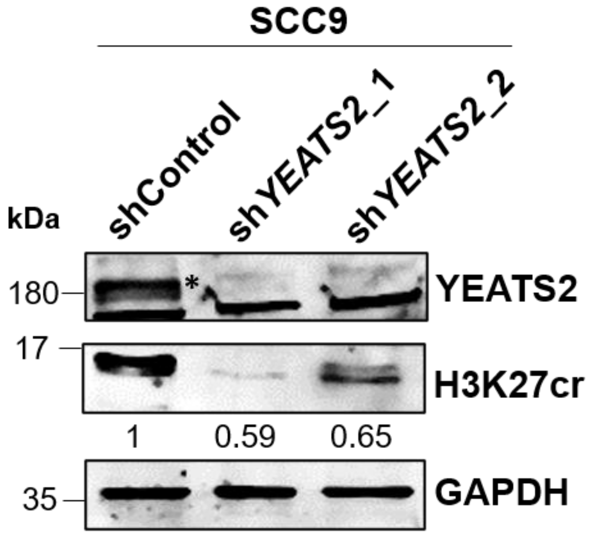

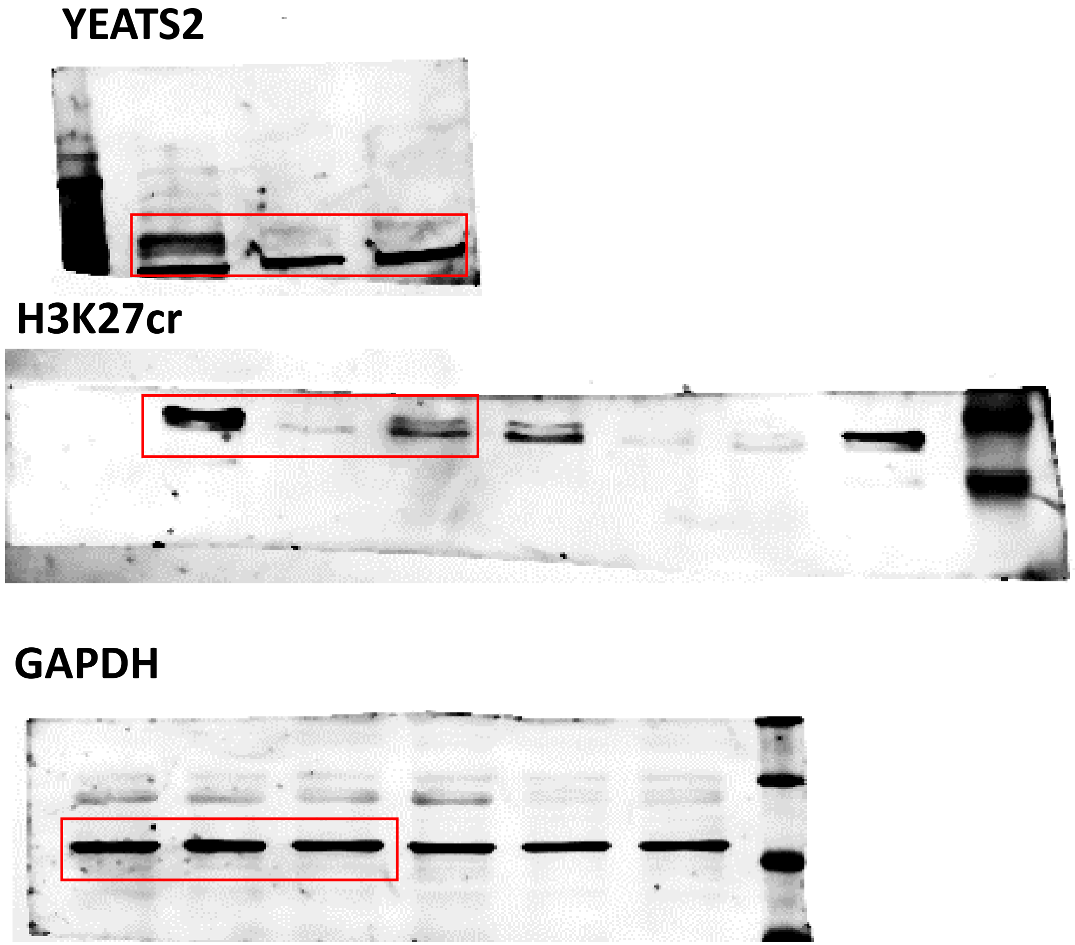

Figure 4- Figure Supplement 4B

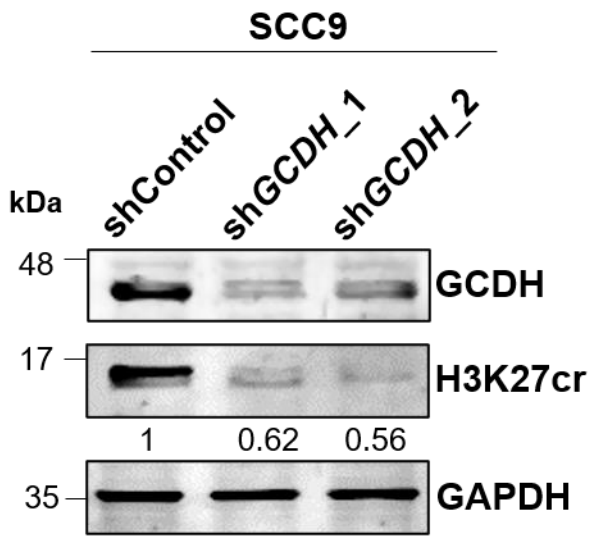

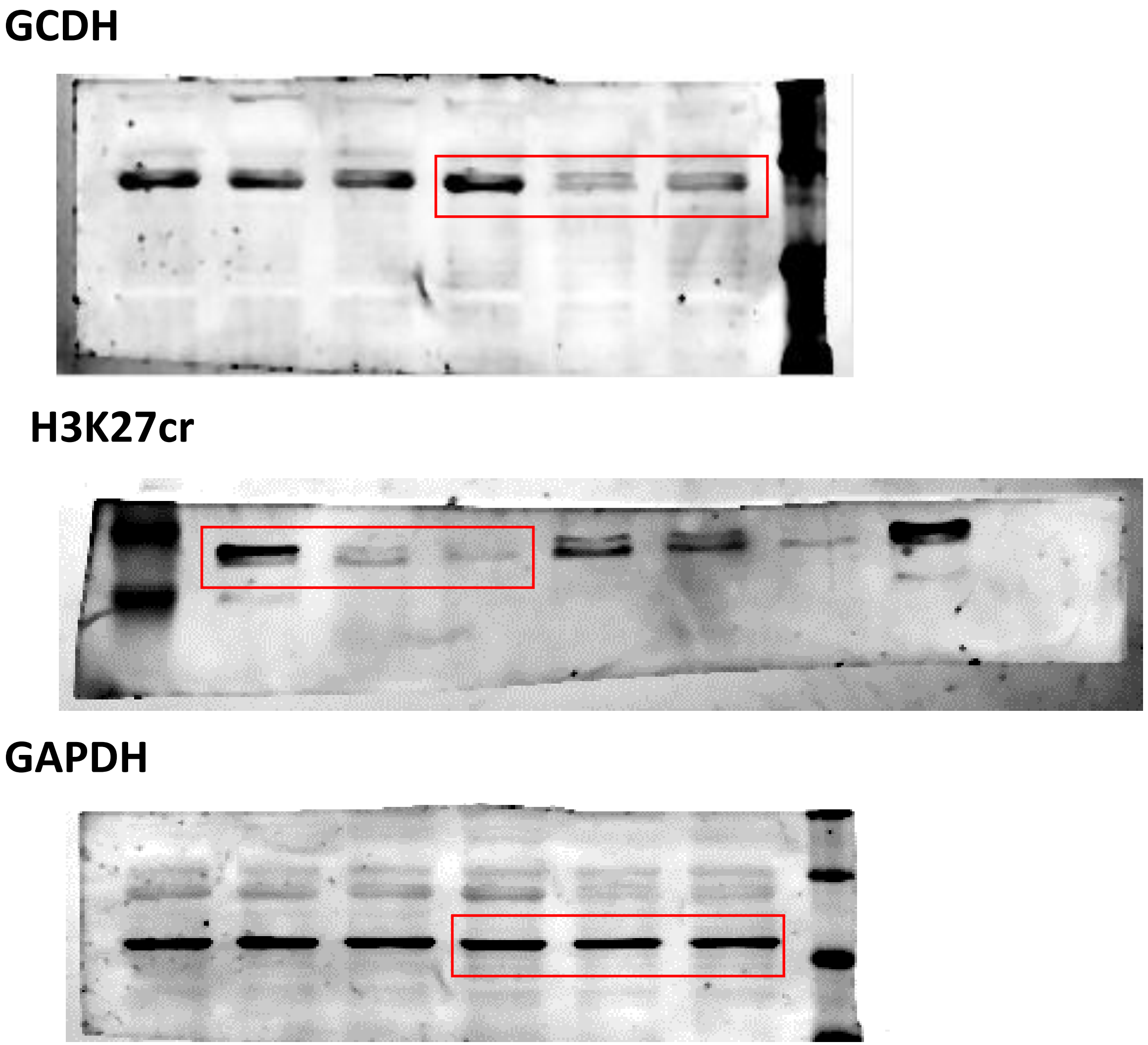

Figure 4—figure supplement 4—Source Data 1. PDF file containing original western blots for Figure 4—figure supplement 4A-B, indicating the relevant bands.
