# Supplementary material for: Interplay of YEATS2 and GCDH regulates histone crotonylation and drives EMT in head and neck cancer
Source: eLife. 2025 Aug 14;14:RP103321. doi: 10.7554/eLife.103321 (PMC12352869; doi:10.7554/eLife.103321)
Supplement: Figure 5—source data 1. [file elife-103321-fig5-data1.zip › Figure 5—Source Data 1/Figure 5E and G-I.pdf]

Figure 5E

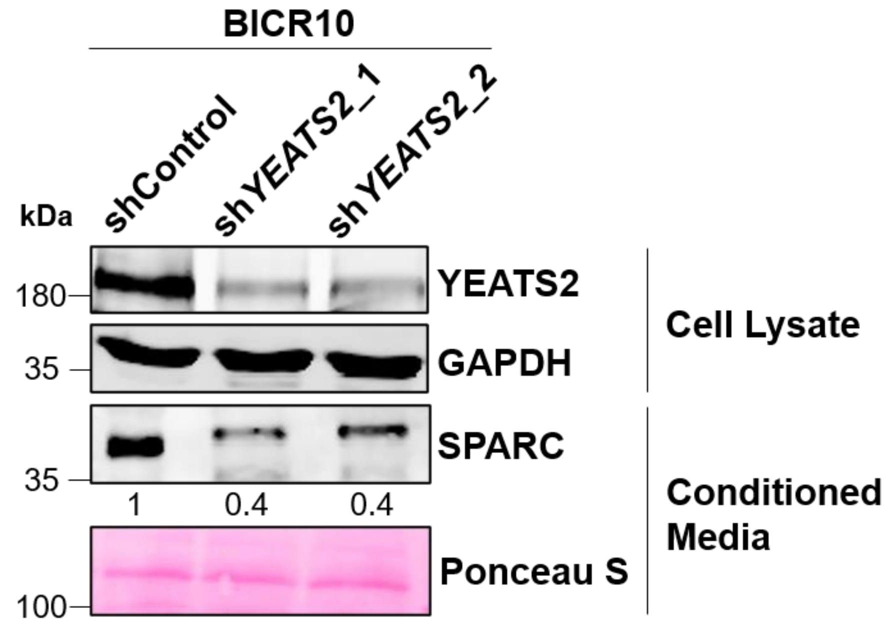
BICR10
shControl
shYEATS2_1
shYEATS2_2
kDa
180
35
35
100
YEATS2
GAPDH
SPARC
Ponceau S
1
0.4
0.4
Cell Lysate
Conditioned Media

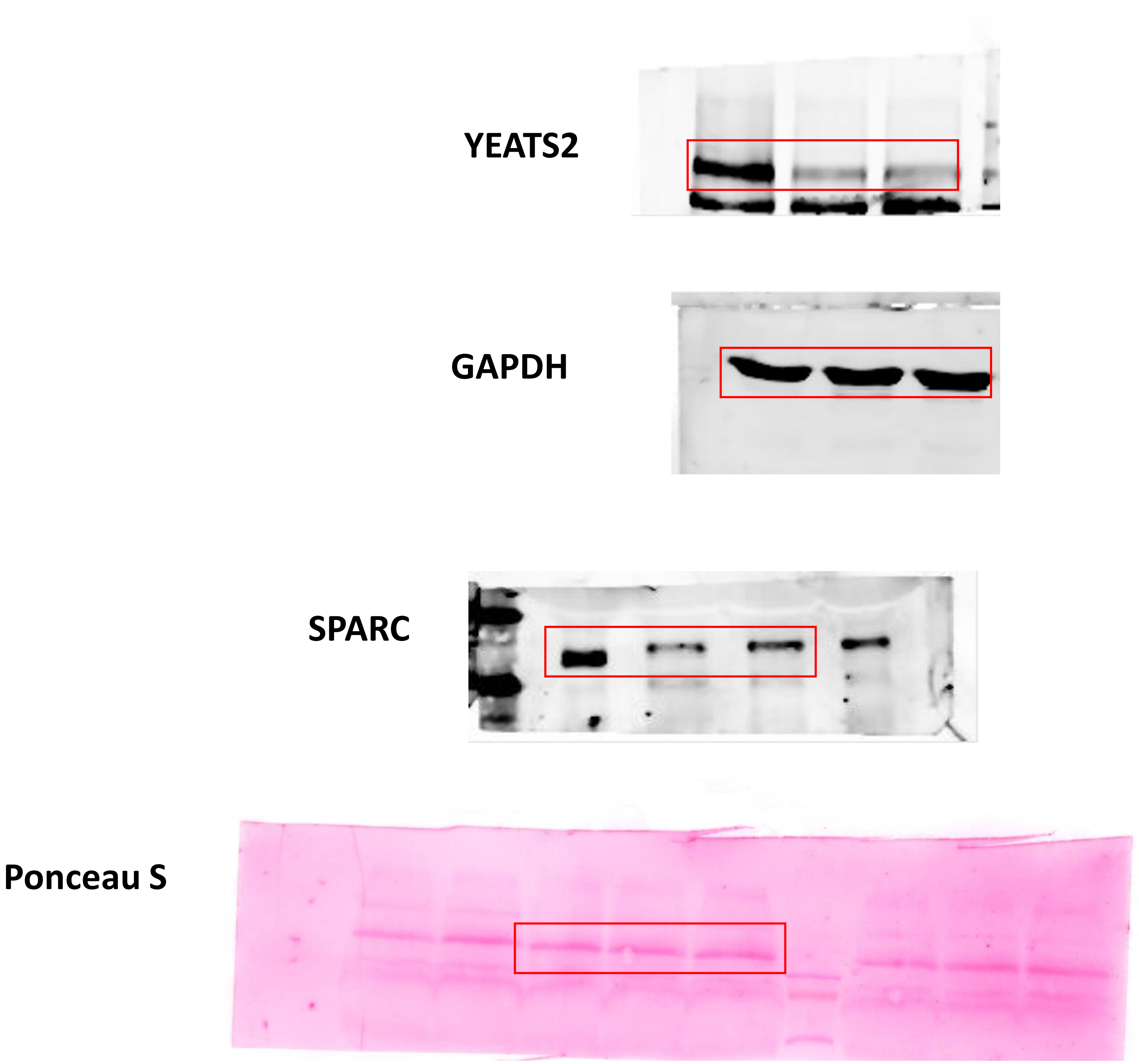
YEATS2
GAPDH
SPARC
Ponceau S

Figure 5G

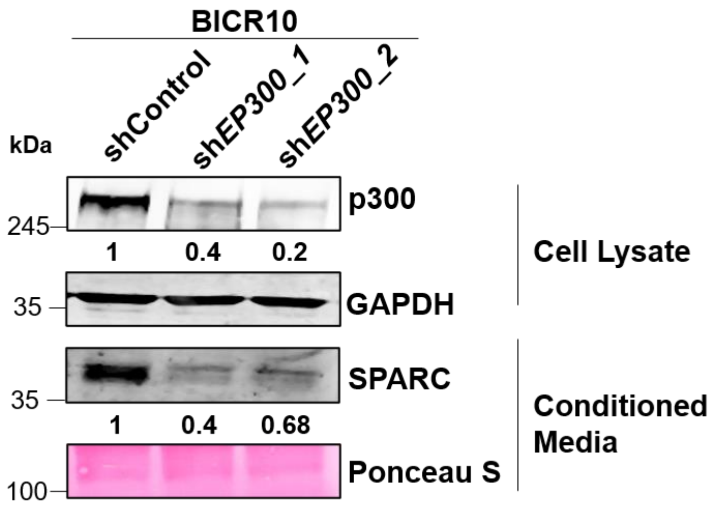

p300

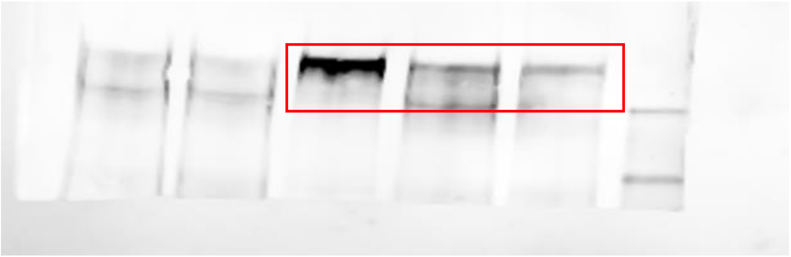

GAPDH

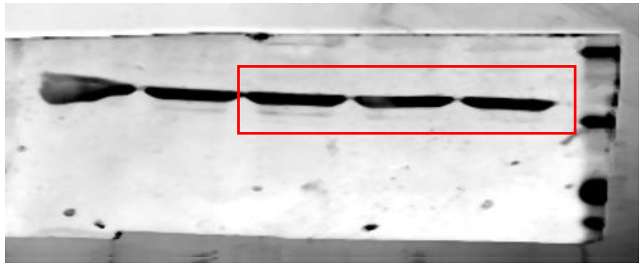

SPARC

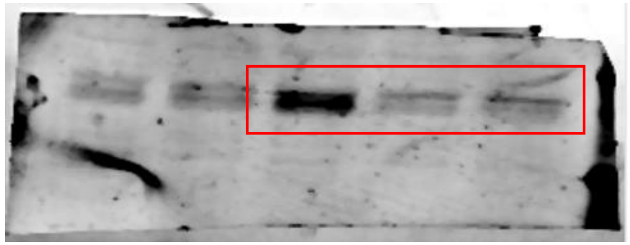

Ponceau S

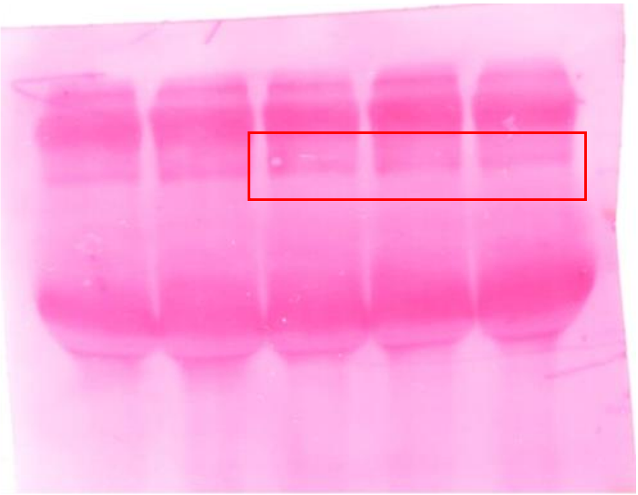

Figure 5H

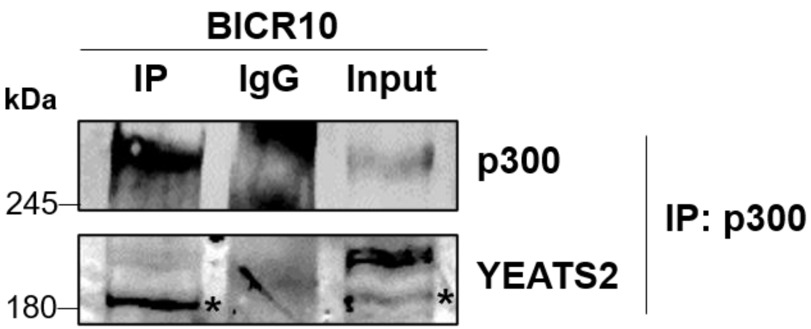

BICR10
IP
IgG
Input
kDa
245
180
p300
YEATS2
*
*
IP: p300

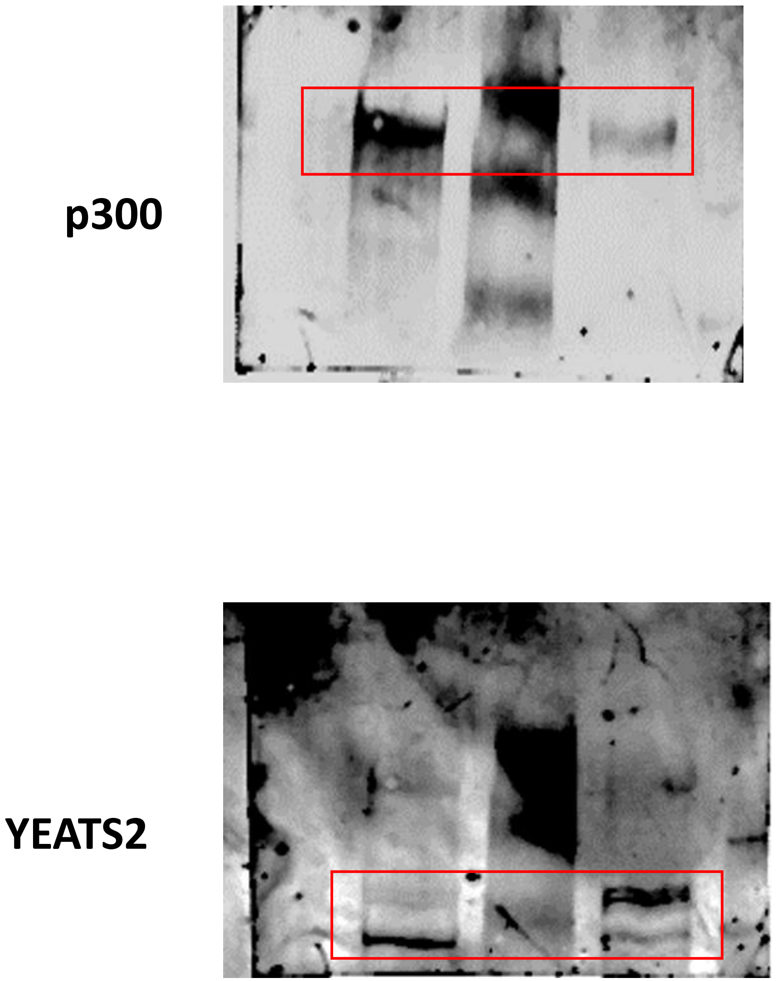

p300
YEATS2

Figure 5I

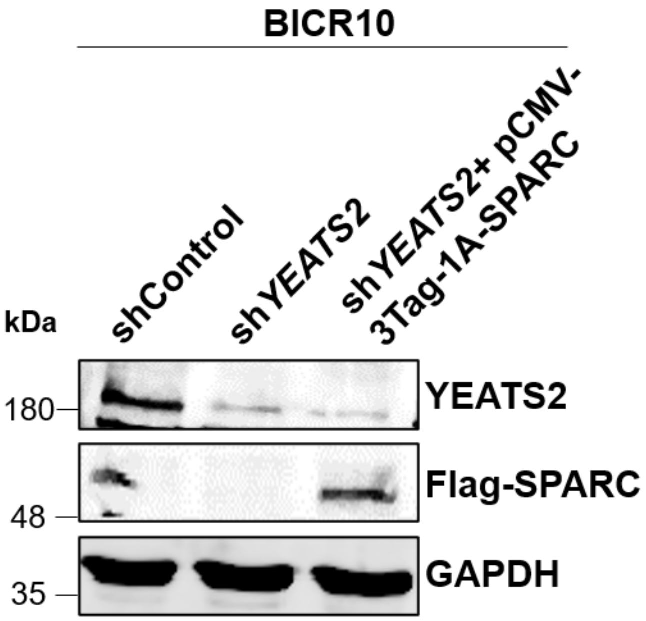

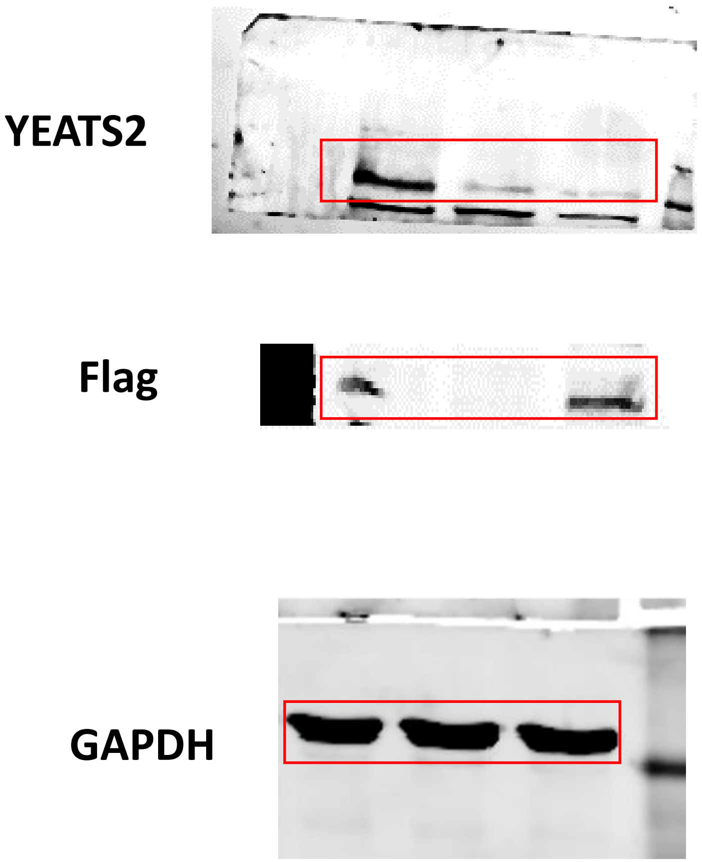

Figure 5—Source Data 1. PDF file containing original western blots for Figure 5E and 5G-I, indicating the relevant bands.
