# Supplementary material for: Interplay of YEATS2 and GCDH regulates histone crotonylation and drives EMT in head and neck cancer
Source: eLife. 2025 Aug 14;14:RP103321. doi: 10.7554/eLife.103321 (PMC12352869; doi:10.7554/eLife.103321)
Supplement: Figure 5—figure supplement 1—source data 1. [file elife-103321-fig5-figsupp1-data1.zip › Figure 5—figure supplement 1—Source Data 1/Figure 5-figure supplement 1F.pdf]

Figure 5- Figure Supplement 1F

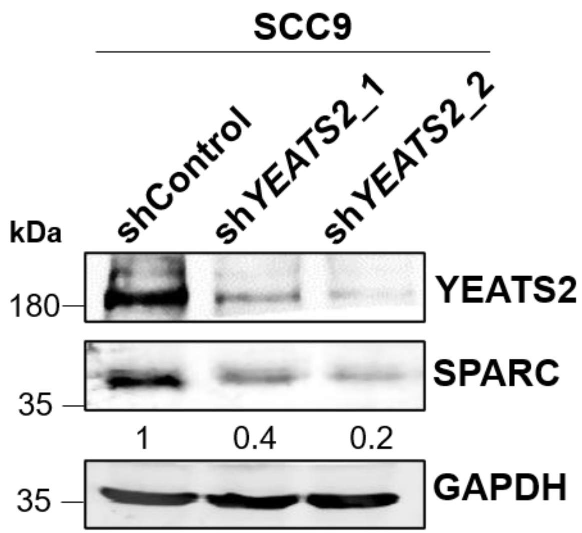

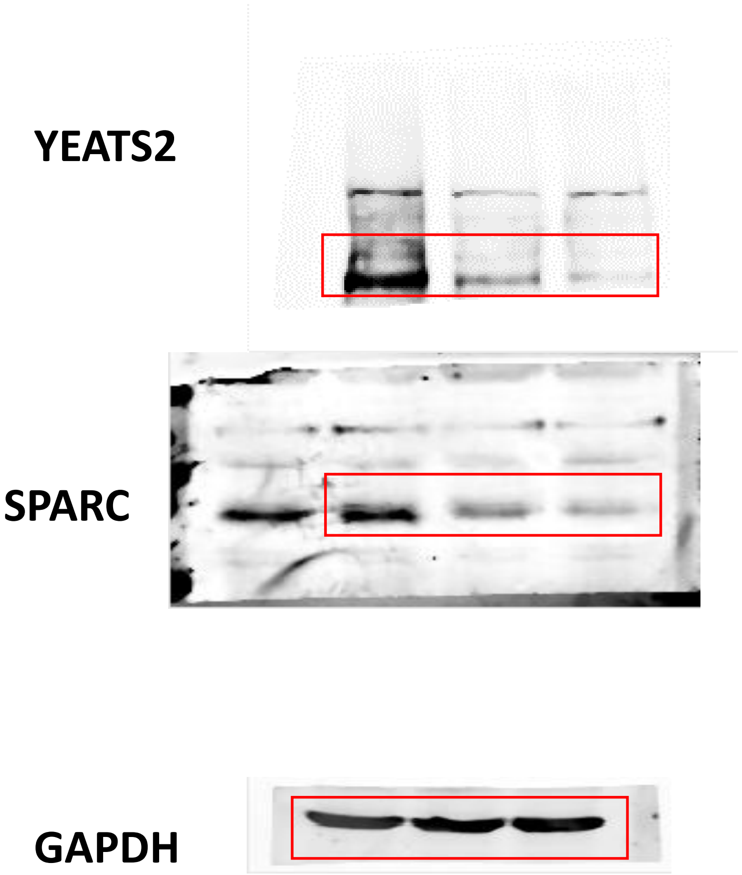

Figure 5—figure supplement 1—Source Data 1. PDF file containing original western blots for Figure 5—figure supplement 1F, indicating the relevant bands.
