# Supplementary material for: Interplay of YEATS2 and GCDH regulates histone crotonylation and drives EMT in head and neck cancer
Source: eLife. 2025 Aug 14;14:RP103321. doi: 10.7554/eLife.103321 (PMC12352869; doi:10.7554/eLife.103321)
Supplement: Figure 7—source data 1. [file elife-103321-fig7-data1.zip › Figure 7—Source Data 1/Figure 7B, 7D and 7H.pdf]

Figure 7B

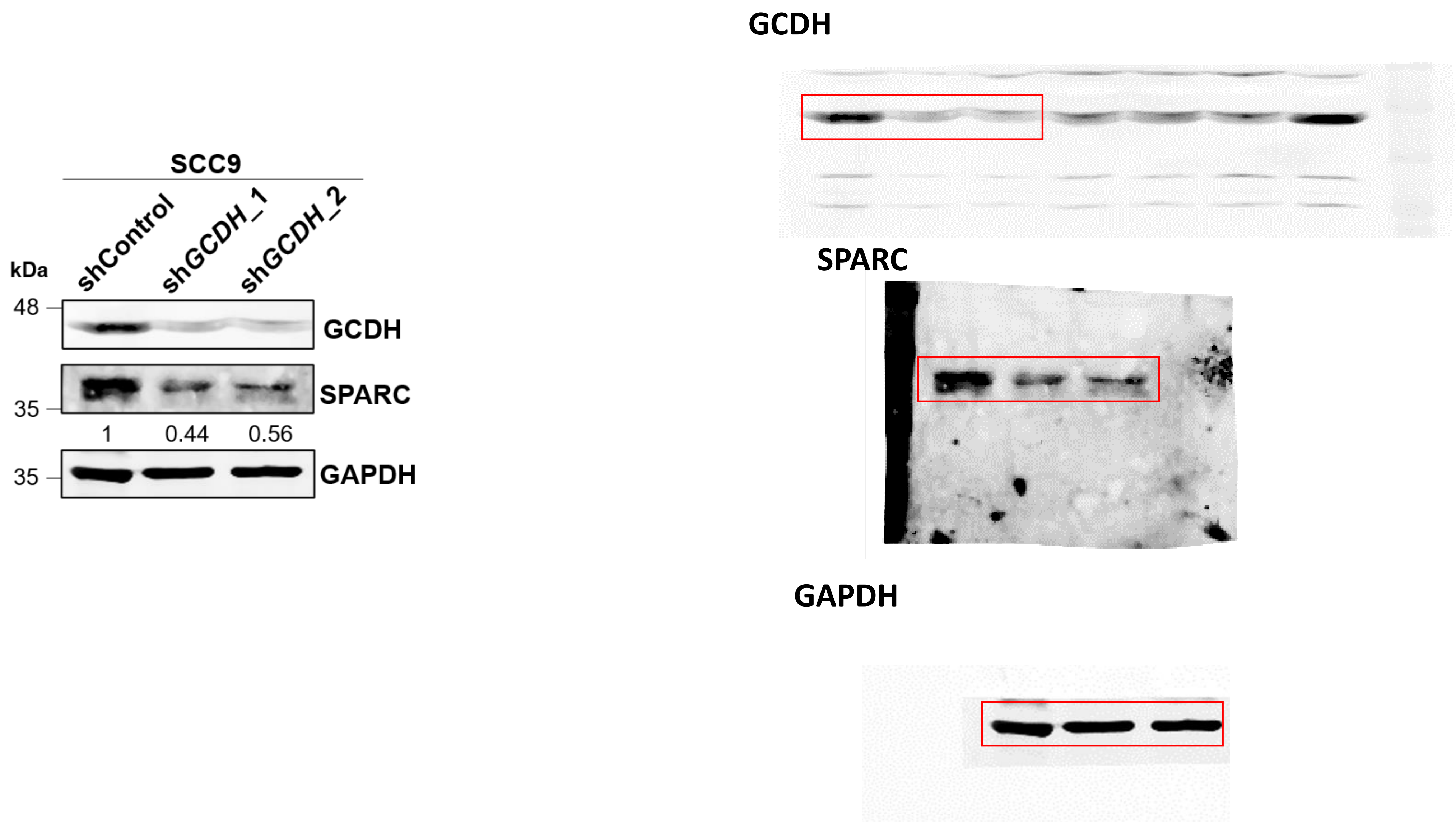
SCC9
shControl
shGCDH_1
shGCDH_2
kDa
48
35
35
GCDH
SPARC
1
0.44
0.56
GAPDH
GCDH
SPARC
GAPDH

Figure 7D

SP1

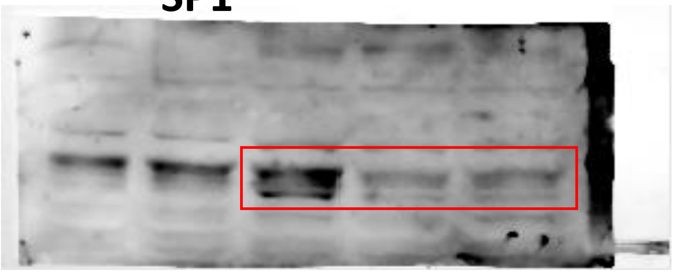

GCDH

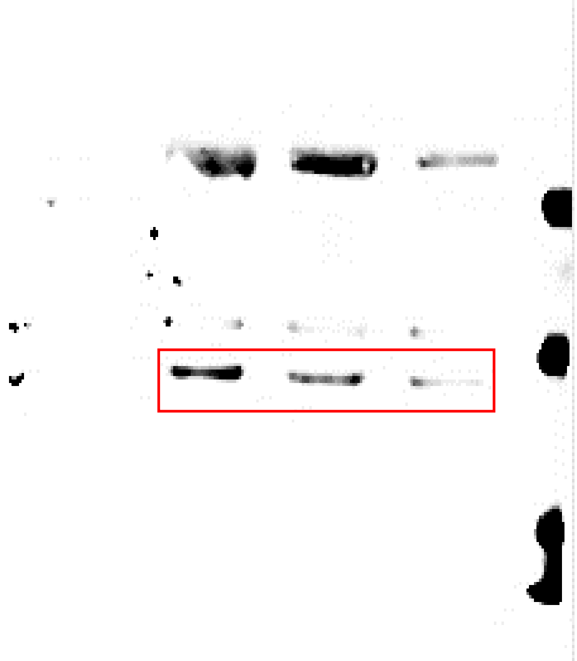

GAPDH

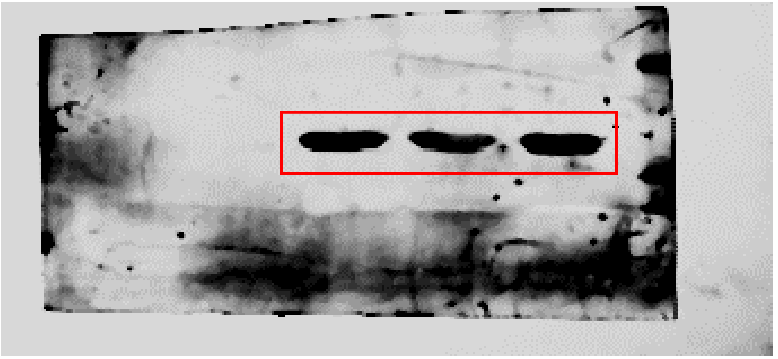

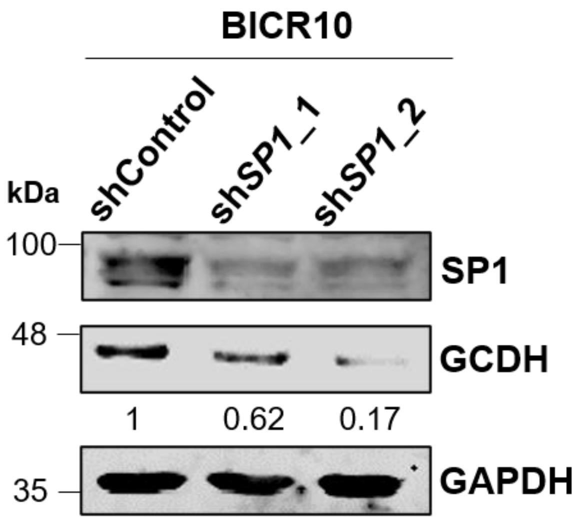

Figure 7H

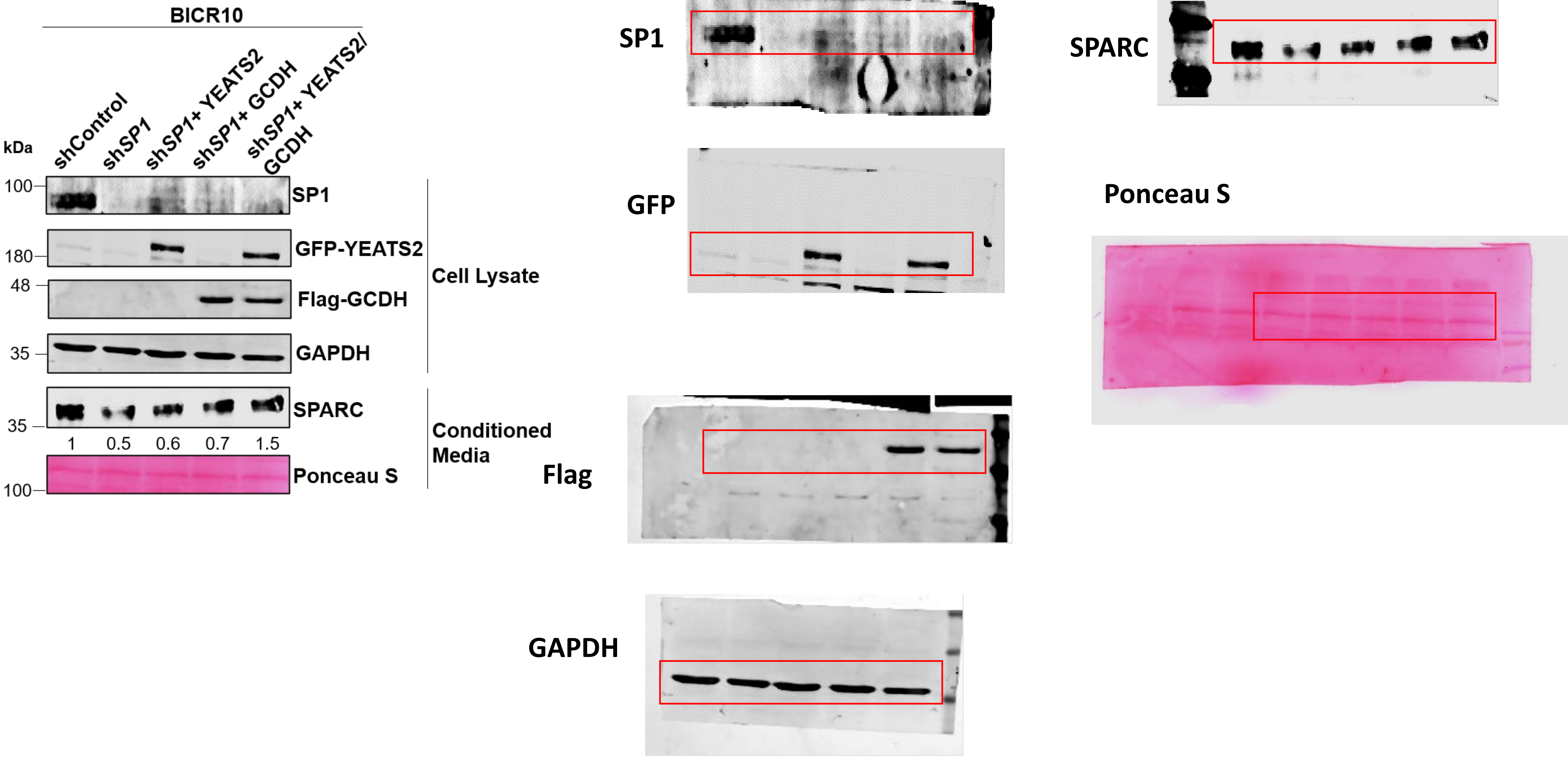

Figure 7—Source Data 1. PDF file containing original western blots for Figure 7B, 7D and 7H, indicating the relevant bands.
