# Supplementary material for: Interplay of YEATS2 and GCDH regulates histone crotonylation and drives EMT in head and neck cancer
Source: eLife. 2025 Aug 14;14:RP103321. doi: 10.7554/eLife.103321 (PMC12352869; doi:10.7554/eLife.103321)
Supplement: Peer review history

# Editor report: eLife Assessment

**DOI:** 10.7554/eLife.103321.3.sa0

**Authors:** Tapas Kumar Kundu

**Affiliations:** Jawaharlal Nehru Centre for Advanced Scientific Research India

**Keywords:** Solid, Useful

These **useful** findings assigned a novel functional implication of histone acylation, crotonylation. Mechanistic insights have been provided in great detail regarding the role of the YEATS2-GCDH axis in modulating epithelial-to-mesenchymal transition (EMT) in head and neck cancer, and overall the strength of evidence is **solid**.

# Referee report: Joint Public Review

**DOI:** 10.7554/eLife.103321.3.sa1

This manuscript investigates a mechanism between the histone reader protein YEATS2 and the metabolic enzyme GCDH, particularly in regulating epithelial-to-mesenchymal transition (EMT) in head and neck cancer (HNC).

The authors addressed most of the concerns of the reviewers. They have:

(1) Increased the patient cohort size from 10 to 23 for evaluating the levels of YEATS2 and H3K27cr.

(2) Checked the expression of major genes involved in the YEATS2-mediated histone crotonylation axis (YEATS2, GCDH, ECHS1, Twist1, along with H3K27cr levels) in head and neck cancer tissues using immunohistochemistry.

(3) Analyzed publicly available head and neck cancer patient datasets, which revealed a significant positive correlation between YEATS2 expression and increasing tumor grade.

(4) Performed GSEA on TCGA HNC patient samples stratified by high versus low YEATS2 expression. This analysis robustly demonstrated a positive enrichment of metastasis-related gene sets in the high YEATS2 expression group, compared to the low YEATS2 group.

(5) Performed extensive experiments to look into the role of p300 in assisting YEATS2 in regulating promoter histone crotonylation. The p300 was knocked down in BICR10 cells, followed by immunoblotting to assess SPARC protein levels.

(6) Performed co-immunoprecipitation assays to check for an interaction between endogenous YEATS2 and p300. The results clearly demonstrate the presence of YEATS2 in the p300-immunoprecipitate sample, indicating that YEATS2 and p300 physically interact and likely function together as a complex to drive the expression of target genes like SPARC.

(7) Performed RNA Polymerase II ChIP-qPCR on the SPARC promoter in YEATS2 knockdown cells.

(8) To confirm p300's specific role in crotonylation at this locus, they performed H3K27cr ChIP-qPCR after p300 knockdown.

(9) Performed SP1 knockdown (which reduces YEATS2 expression) followed by ectopic YEATS2 overexpression, and then assessed p300 occupancy and H3K27cr levels on the SPARC promoter.

# Author comment: Author response

**DOI:** 10.7554/eLife.103321.3.sa2

**Authors:** Deepak Pant, PARIK KAKANI, Rushikesh Joshi, Abin Sabu, Shruti Agrawal, Atul Samaiya, Sanjeev Shukla

**Affiliations:** Indian Institute of Science Education and Research, Bhopal Bhopal India; Indian Institute of Science Education and Research Bhopal India; Indian Institute of Science Education and Research, Bhopal Bhopal India; Indian Institute of Science Education and Research, Bhopal Bhopal India; Bansal Hospital Bhopal India; Bansal Hospital Bhopal India; Indian Institute of Science Education and Research, Bhopal Bhopal India

The following is the authors’ response to the original reviews.

We sincerely appreciate the editors for overseeing an efficient review process and for upholding the high standards of the journal. We have made extensive revisions to the manuscript after carefully reviewing the reviewers’ comments. We have addressed all the comments in our response and have incorporated the changes suggested by the reviewers to the best of our abilities. Notably, we have made the following major changes to the manuscript:

(1) We have increased the patient cohort size from 10 to 23 for evaluating the levels of YEATS2 and H3K27cr.

(2) To further strengthen the clinical relevance of our study, we have checked the expression of major genes involved in the YEATS2-mediated histone crotonylation axis (YEATS2, GCDH, ECHS1, Twist1 along with H3K27cr levels) in head and neck cancer tissues using immunohistochemistry.

(3) We have performed extensive experiments to look into the role of p300 in assisting YEATS2 in regulating promoter histone crotonylation.

The changes made to the manuscript figures have been highlighted in our response. We have also updated the Results section in accordance with the updated figures. Tables 1-4 and Supplementary files 1-3 have been moved to one single Excel workbook named ‘Supplementary Tables 1-8’. Additional revisions have been made to improve the overall quality of the manuscript and enhance data visualization. These additional changes are highlighted in the tracked changes version of the manuscript.

Our response to the Public Reviews and ‘Recommendations to the Authors’ can be found below.

**Public Reviews:**

**Reviewer #1 (Public review):**

Summary:

This manuscript investigates a mechanism between the histone reader protein YEATS2 and the metabolic enzyme GCDH, particularly in regulating epithelial-to-mesenchymal transition (EMT) in head and neck cancer (HNC).

Strengths:

Great detailing of the mechanistic aspect of the above axis is the primary strength of the manuscript.

Weaknesses:

Several critical points require clarification, including the rationale behind EMT marker selection, the inclusion of metastasis data, the role of key metabolic enzymes like ECHS1, and the molecular mechanisms governing p300 and YEATS2 interactions.

We would like to sincerely thank the reviewer for the detailed, in-depth, and positive response. We have implemented constructive revisions to the manuscript to address the reviewer’s concerns effectively.

Major Comments:

(1) The title, "Interplay of YEATS2 and GCDH mediates histone crotonylation and drives EMT in head and neck cancer," appears somewhat misleading, as it implies that YEATS2 directly drives histone crotonylation. However, YEATS2 functions as a reader of histone crotonylation rather than a writer or mediator of this modification. It cannot itself mediate the addition of crotonyl groups onto histones. Instead, the enzyme GCDH is the one responsible for generating crotonyl-CoA, which enables histone crotonylation. Therefore, while YEATS2 plays a role in recognizing crotonylation marks and may regulate gene expression through this mechanism, it does not directly catalyse or promote the crotonylation process.

We thank the reviewer for their insightful comment regarding the precision of our title. We agree that the initial wording 'mediates' could imply a direct enzymatic role for YEATS2 in histone crotonylation, which is indeed not the case. As the reviewer correctly points out, YEATS2 functions as a 'reader' of histone crotonylation marks.

However, our research demonstrates that YEATS2 plays a crucial indirect regulatory role in the establishment of these crotonylation marks. Specifically, our data indicates that YEATS2 facilitates the recruitment of the histone crotonyltransferase p300 to specific gene promoters, such as that of *SPARC*. This recruitment mechanism directly impacts the localized deposition of crotonyl marks on nearby histone residues. Therefore, while YEATS2 does not directly catalyze the addition of crotonyl groups, its presence and interaction with p300 are essential for the regulation and establishment of histone crotonylation at these critical sites.

To accurately reflect this nuanced, yet significant, regulatory mechanism, we have revised the title. We are replacing 'mediates' with 'regulates' to precisely convey that YEATS2 influences the histone crotonylation process, albeit indirectly, through its role in recruiting the enzymatic machinery. The updated title will now read: 'Interplay of YEATS2 and GCDH regulates histone crotonylation and drives EMT in head and neck cancer.' We believe this change maintains the core message of our findings while enhancing the scientific accuracy of the title.

(2) The study suggests a link between YEATS2 and metastasis due to its role in EMT, but the lack of clinical or pre-clinical evidence of metastasis is concerning. Only primary tumor (PT) data is shown, but if the hypothesis is that YEATS2 promotes metastasis via EMT, then evidence from metastatic samples or in vivo models should be included to solidify this claim.

We thank the reviewer for their valuable suggestion regarding the need for clinical or pre-clinical evidence of metastasis. We fully agree that direct evidence linking YEATS2 to metastasis would significantly strengthen our claims, especially given its demonstrated role in EMT.

Our primary objective in this study was to meticulously dissect the molecular mechanisms by which YEATS2 regulates histone crotonylation and drives EMT in head and neck cancer. We have provided comprehensive upstream and downstream molecular insights into this process, culminating in a clear demonstration of YEATS2's functional importance in promoting EMT through multiple in vitro phenotypic assays (e.g., Matrigel invasion, wound healing, 3D invasion assays). As the reviewer notes, EMT is a widely recognized prerequisite for cancer metastasis[1]. Therefore, establishing YEATS2 as a driver of EMT directly implicates its potential role in metastatic progression.

To further address the reviewer's concern and bridge the gap between EMT and metastasis, we have performed additional analyses that will be incorporated into the revised manuscript:

Clinical Correlation with Tumor Grade: We analyzed publicly available head and neck cancer patient datasets. Our analysis revealed a significant positive correlation between YEATS2 expression and increasing tumor grade. Specifically, we observed significantly higher YEATS2 expression in Grade 2-4 tumors compared to Grade 1 tumors. Given that higher tumor grades are frequently associated with increased metastatic potential and poorer prognosis in HNC[2], this finding provides compelling clinical correlative evidence linking elevated YEATS2 expression to more aggressive disease.

Gene Set Enrichment Analysis (GSEA) for Metastasis Pathways: To further explore the biological processes associated with YEATS2 in a clinical context, we performed GSEA on TCGA HNC patient samples stratified by high versus low YEATS2 expression. This analysis robustly demonstrated a positive enrichment of metastasis-related gene sets in the high YEATS2 expression group, compared to the low YEATS2 group. This strengthens the mechanistic link by showing that pathways associated with metastasis are co-ordinately upregulated when YEATS2 is highly expressed.

These new clinical data provide strong correlative evidence supporting a direct association of YEATS2 with metastasis, building upon our detailed mechanistic dissection of its role in EMT.

(3) There seems to be some discrepancy in the invasion data with BICR10 control cells (Figure 2C). BICR10 control cells with mock plasmids, specifically shControl and pEGFP-C3 show an unclear distinction between invasion capacities. Normally, we would expect the control cells to invade somewhat similarly, in terms of area covered, within the same time interval (24 hours here). But we clearly see more control cells invading when the invasion is done with KD and fewer control cells invading when the invasion is done with OE. Are these just plasmid-specific significant effects on normal cell invasion? This needs to be addressed.

We thank the reviewer for their careful examination of Figure 2C and their insightful observation regarding the appearance of the control cells in relation to the knockdown (Figure 2B) and overexpression (Figure 2C) experiments. We understand how, at first glance, the control invasion levels across these panels might seem disparate.

We wish to clarify that Figure 2B (YEATS2 knockdown) and Figure 2C (YEATS2 overexpression) represent two entirely independent experiments, conducted with distinct experimental conditions and methodologies, as detailed in our Methods section.

Specifically:

Figure 2B (Knockdown): Utilizes lentivirus-mediated transduction for stable shRNA delivery (shControl as control).

Figure 2C (Overexpression): Utilizes transfection with plasmid DNA (pEGFP-C3 as control) via a standard transfection reagent.

These fundamental differences in genetic manipulation methods (transduction vs. transfection), along with potential batch-to-batch variations in reagents or cell passage number at the time of each independent experiment, can indeed lead to variations in absolute basal invasion rates of control cells[3].

Therefore, the invasion capacity of BICR10 control cells in Figure 2B (shControl) should only be compared to the YEATS2 knockdown conditions within that same panel. Similarly, the invasion capacity of control cells in Figure 2C (pEGFP-C3) should only be compared to the YEATS2 overexpression conditions within that specific panel. The crucial finding in each panel lies in the relative change in invasion caused by YEATS2 manipulation (knockdown or overexpression) compared to its respective, concurrently run control.

We have ensured that all statistical analyses (as indicated in the figure legends and methods) were performed by comparing the experimental groups directly to their matched internal controls within each independent experiment. The significant increase in invasion upon YEATS2 overexpression and the significant decrease upon YEATS2 knockdown, relative to their respective controls, are robust and reproducible findings.

(4) In Figure 3G, the Western blot shows an unclear band for YEATS2 in shSP1 cells with YEATS2 overexpression condition. The authors need to clearly identify which band corresponds to YEATS2 in this case.

We thank the reviewer for pointing out the ambiguity in the YEATS2 Western blot for the shSP1 + pEGFP-C3-YEATS2 condition in Figure 3G. We apologize for this lack of clarity. The two bands seen in the shSP1+pEGFP-C3-YEATS2 condition correspond to the endogenous YEATS2 band (lower band) and YEATS2-GFP band (upper band, corresponding to overexpressed YEATS2-GFP fusion protein, which has a higher molecular weight). To avoid confusion, the endogenous band is now highlighted (marked by *) in the lane representing the shSP1+pEGFP-C3-YEATS2 condition. We have also updated the figure legend accordingly.

(5) In ChIP assays with SP1, YEATS2 and p300 which promoter regions were selected for the respective genes? Please provide data for all the different promoter regions that must have been analysed, highlighting the region where enrichment/depletion was observed. Including data from negative control regions would improve the validity of the results.

Throughout our study, we have performed ChIP-qPCR assays to check the binding of SP1 on *YEATS2* and *GCDH* promoter, and to check YEATS2 and p300 binding on *SPARC* promoter. Using transcription factor binding prediction tools and luciferase assays, we selected multiple sites on the *YEATS2* and *GCDH* promoter to check for SP1 binding. The results corresponding to the site that showed significant enrichment were provided in the manuscript. The region of *SPARC* promoter in YEATS2 and p300 ChIP assay was selected on the basis of YEATS2 enrichment found in the YEATS2 ChIP-seq data. The ChIP-qPCR data for all the promoter regions investigated (including negative controls) can be found below (Author response image 1).

Author response image 1

**Author response image 1.** 

(A) SP1 ChIP-qPCR results indicating SP1 occupancy on different regions of *YEATS2* promoter. *YEATS2* promoter region showing SP1 binding sites (indicated by red boxes) is shown above. SP1 showed significant enrichment at F1R1 region. The results corresponding to F1R1 region were included in Figure 3D. (B) SP1 ChIPqPCR results indicating SP1 occupancy on different regions of *GCDH* promoter. *GCDH* promoter region showing SP1 binding sites (indicated by red boxes) is shown above. SP1 showed significant enrichment at F2R2 region. The results corresponding to F2R2 region were included in Figure 7E. (C) YEATS2 ChIP-qPCR results in shControl vs. sh*YEATS2* BICR10 cells indicating YEATS2 occupancy on different regions of *SPARC* promoter. *SPARC* promoter region showing YEATS2 ChIP-seq and H3K27cr ChIP-seq signals is shown above. YEATS2 showed significant enrichment at F1R1 region. The results corresponding to F1R1 region were included in Figure 5C. (D) p300 ChIP-qPCR results in shControl vs. sh*YEATS2* BICR10 cells indicating p300 occupancy on different regions of *SPARC* promoter. p300 showed significant enrichment at F1R1 region. The results corresponding to F1R1 region were included in Figure 5F.

(6) The authors establish a link between H3K27Cr marks and GCDH expression, and this is an already well-known pathway. A critical missing piece is the level of ECSH1 in patient samples. This will clearly delineate if the balance shifted towards crotonylation.

We greatly appreciate the reviewer's insightful comment regarding the importance of assessing ECSH1 levels in patient samples to clearly delineate the metabolic balance shifting towards crotonylation. We fully agree that this is a critical piece of evidence.

To directly address this point and substantiate our claim regarding the altered metabolic balance in HNC, we had previously analyzed the expression of both GCDH and ECHS1 in TCGA HNC RNA-seq data (as presented in Figure 4—figure supplement 1A and B). This analysis revealed a consistent increase in GCDH expression and a concomitant decrease in ECHS1 expression in tumor samples compared to normal tissues. Based on these findings, we hypothesized that this altered expression profile would indeed lead to an accumulation of crotonyl-CoA and, consequently, an overall increase in histone crotonylation in HNC.

To further validate and extend these findings at the protein level, we have now performed immunohistochemistry (IHC) analysis for both ECHS1 and GCDH in a cohort of HNC normal vs. tumor tissues. Our IHC results strikingly corroborate the RNA-seq data: GCDH consistently showed increased protein expression in tumor samples, whereas ECHS1 exhibited significantly reduced protein expression in tumors compared to their adjacent normal counterpart tissues (Figure 4E).

These new data, combined with existing TCGA HNC RNA-seq analysis strongly supports our proposed mechanism where altered GCDH and ECHS1 expression contributes to increased histone crotonylation in head and neck cancer.

(7) The p300 ChIP data on the SPARC promoter is confusing. The authors report reduced p300 occupancy in YEATS2-silenced cells, on SPARC promoter. However, this is paradoxical, as p300 is a writer, a histone acetyltransferase (HAT). The absence of a reader (YEATS2) shouldn't affect the writer (p300) unless a complex relationship between p300 and YEATS2 is present. The role of p300 should be further clarified in this case. Additionally, transcriptional regulation of SPARC expression in YEATS2 silenced cells could be analysed via downstream events, like Pol-II recruitment. Assays such as Pol-II ChIP-qPCR could help explain this.

We greatly appreciate the reviewer's insightful observation regarding the apparently paradoxical reduction of p300 occupancy on the SPARC promoter upon YEATS2 silencing (Figure 5F), and their call for further clarification of p300's role and the potential complex relationship with YEATS2. We agree that this point required further mechanistic investigation.

As we have shown through RNA-seq and ChIP-seq analyses, YEATS2 broadly influences histone crotonylation levels at gene promoters, thereby impacting gene expression. While p300 is indeed a known histone acetyltransferase (HAT) with promiscuous acyltransferase activity, including crotonyltransferase activity[4], the precise mechanism by which its occupancy is affected by a 'reader' protein like YEATS2 was unclear. Our initial data suggested a dependency of p300 recruitment on YEATS2.

To directly address the reviewer's concern and thoroughly delineate the molecular mechanism of cooperativity between YEATS2 and p300 in regulating histone crotonylation, we have now performed a series of targeted experiments, which have been incorporated into the revised manuscript:

(a) Validation of p300's role in SPARC expression: We performed p300 knockdown in BICR10 cells, followed by immunoblotting to assess SPARC protein levels. As expected, a significant decrease in SPARC protein levels was observed upon p300 knockdown (Figure 5G). This confirms p300's direct involvement in SPARC gene expression.

(b) Direct interaction between YEATS2 and p300: To investigate a potential physical association, we performed co-immunoprecipitation assays to check for an interaction between endogenous YEATS2 and p300. Our results clearly demonstrate the presence of YEATS2 in the p300-immunoprecipitate sample, indicating that YEATS2 and p300 physically interact and likely function together as a complex to drive the expression of target genes like *SPARC* (Figure 5H). This direct interaction provides the mechanistic basis for how YEATS2 influences p300 occupancy.

(c) Impact on transcriptional activity (Pol II recruitment): As suggested, we performed RNA Polymerase II (Pol II) ChIP-qPCR on the *SPARC* promoter in YEATS2 knockdown cells. We observed a significant decrease in Pol II occupancy on the *SPARC* promoter after YEATS2 knockdown in BICR10 cells (Figure 6C). This confirms that YEATS2 silencing leads to reduced transcriptional initiation/elongation at this promoter.

(d) p300's direct role in H3K27cr on *SPARC* promoter: To confirm p300's specific role in crotonylation at this locus, we performed H3K27cr ChIP-qPCR after p300 knockdown. As anticipated, a significant decrease in H3K27cr enrichment was observed on the *SPARC* promoter upon p300 knockdown (Figure 6J), directly demonstrating p300's crotonyltransferase activity at this site.

(e) Rescue of p300 occupancy and H3K27cr by YEATS2 overexpression in SP1deficient cells: To further establish the YEATS2-p300 axis, we performed SP1 knockdown (which reduces YEATS2 expression) followed by ectopic YEATS2 overexpression, and then assessed p300 occupancy and H3K27cr levels on the *SPARC* promoter. While SP1 knockdown led to a decrease in both p300 and H3K27cr enrichment, we observed a significant rescue of both p300 occupancy and H3K27cr enrichment upon YEATS2 overexpression in the shSP1 cells (Figure 6K and L). This provides strong evidence that YEATS2 acts downstream of SP1 to regulate p300 recruitment and H3K27cr levels.

Collectively, these comprehensive new results clearly establish that YEATS2 directly interacts with and assists in the recruitment of p300 to the *SPARC* promoter. This recruitment is crucial for p300's localized crotonyltransferase activity, leading to increased H3K27cr marks and subsequent activation of *SPARC* transcription. This clarifies the previously observed 'paradox' and defines a novel cooperative mechanism between a histone reader (YEATS2) and a writer (p300) in regulating histone crotonylation and gene expression.

(8) The role of GCDH in producing crotonyl-CoA is already well-established in the literature. The authors' hypothesis that GCDH is essential for crotonyl-CoA production has been proven, and it's unclear why this is presented as a novel finding. It has been shown that YEATS2 KD leads to reduced H3K27cr, however, it remains unclear how the reader is affecting crotonylation levels. Are GCDH levels also reduced in the YEATS2 KD condition? Are YEATS2 levels regulating GCDH expression? One possible mechanism is YEATS2 occupancy on GCDH promoter and therefore reduced GCDH levels upon YEATS2 KD. This aspect is crucial to the study's proposed mechanism but is not addressed thoroughly.

We appreciate the reviewer's valuable comment questioning the novelty of GCDH's role in crotonyl-CoA production and seeking further clarification on how YEATS2 influences crotonylation levels beyond its reader function.

We agree that GCDH's general role in producing crotonyl-CoA is well-established[5,6]. Our study, however, aims to delineate a novel epigenetic-metabolic crosstalk in head and neck cancer, specifically investigating how the interplay between the histone crotonylation reader YEATS2 and the metabolic enzyme GCDH contributes to increased histone crotonylation and drives EMT in this context.

Our initial investigations using GSEA on publicly available TCGA RNA-seq data revealed that HNC patients with high YEATS2 expression also exhibit elevated expression of genes involved in the lysine degradation pathway, prominently including GCDH. Recognizing the known roles of YEATS2 in preferentially binding H3K27cr7 and GCDH in producing crotonylCoA, we hypothesized that the elevated H3K27cr levels observed in HNC are a consequence of the combined action of both YEATS2 and GCDH. We have provided evidence that increased nuclear GCDH correlates with higher H3K27cr abundance, likely due to an increased nuclear pool of crotonyl-CoA, and that YEATS2 contributes through its preferential maintenance of crotonylation marks by recruiting p300 (as detailed in Figure 5F-H and Figure 6J-L of the manuscript and elaborated in our response to point 7). Thus, our work highlights that both YEATS2 and GCDH are crucial for the regulation of histone crotonylation-mediated gene expression in HNC.

To directly address the reviewer's query regarding YEATS2's influence on GCDH levels and nuclear histone crotonylation:

• YEATS2 does not transcriptionally regulate GCDH: We did not find any evidence of YEATS2 directly regulating the expression levels of GCDH at the transcriptional level in HNC cells.

• Novel finding: YEATS2 regulates GCDH nuclear localization: Crucially, we discovered that YEATS2 downregulation significantly reduces the nuclear pool of GCDH in head and neck cancer cells (Figure 7G). This is a novel mechanism suggesting that YEATS2 influences histone crotonylation not only by affecting promoter H3K27cr levels via p300 recruitment, but also by regulating the availability of the crotonyl-CoA producing enzyme, GCDH, within the nucleus.

• Common upstream regulation by SP1: Interestingly, we found that both YEATS2 and GCDH expression are commonly regulated by the transcription factor SP1 in HNC. Our data demonstrate that SP1 binds to the promoters of both genes, and its downregulation leads to a decrease in their respective expressions (Figure 3 and Figure 7). This provides an important upstream regulatory link between these two key players.

• Functional validation of GCDH in EMT: We further assessed the functional importance of GCDH in maintaining the EMT phenotype in HNC cells. Matrigel invasion assays after GCDH knockdown and overexpression in BICR10 cells revealed that the invasiveness of HNC cells was significantly reduced upon GCDH knockdown and significantly increased upon GCDH overexpression (results provided in revised manuscript Figure 7F and Figure 7—figure supplement 1F).

These findings collectively demonstrate a multifaceted role for YEATS2 in regulating histone crotonylation by both direct recruitment of the writer p300 and by influencing the nuclear availability of the crotonyl-CoA producing enzyme GCDH. We acknowledge that the precise molecular mechanism governing YEATS2's effect on GCDH nuclear localization remains an exciting open question for future investigation, but our current data establishes a novel regulatory axis.

(9) The authors should provide IHC analysis of YEATS2, SPARC alongside H3K27cr and GCDH staining in normal vs. tumor tissues from HNC patients.

We thank the reviewer for their suggestion. We have performed IHC analysis for YEATS2, H3K27cr and GCDH in normal and tumor samples obtained from HNC patients.

**Reviewer #2 (Public review):**

Summary:

The manuscript emphasises the increased invasive potential of histone reader YEATS2 in an SP1-dependent manner. They report that YEATS2 maintains high H3K27cr levels at the promoter of EMT-promoting gene SPARC. These findings assigned a novel functional implication of histone acylation, crotonylation.

We thank the reviewer for the constructive comments. We are committed to making beneficial changes to the manuscript in order to alleviate the reviewer’s concerns.

Concerns:

(1) The patient cohort is very small with just 10 patients. To establish a significant result the cohort size should be increased.

We thank the reviewer for this suggestion. We have increased the number of patient samples to assess the levels of YEATS2 (n=23 samples) and the results have been included in Figure 1G and Figure 1—figure supplement 1F.

(2) Figure 4D compares H3K27Cr levels in tumor and normal tissue samples. Figure 1G shows overexpression of YEATS2 in a tumor as compared to normal samples. The loading control is missing in both. Loading control is essential to eliminate any disparity in protein concentration that is loaded.

To address the reviewer’s concern, we have repeated the experiment and used H3 as a loading control as nuclear protein lysates from patient samples were used to check YEATS2 and H3K27cr levels.

(3) Figure 4D only mentions 5 patient samples checked for the increased levels of crotonylation and hence forms the basis of their hypothesis (increased crotonylation in a tumor as compared to normal). The sample size should be more and patient details should be mentioned.

As part of the revision, we have now checked the H3K27cr levels in a total of 23 patient samples and the results have been included in Figure 4D and Figure 4— figure supplement 1D. Patient details are provided in Supplementary file 6.

(4) YEATS2 maintains H3K27Cr levels at the SPARC promoter. The p300 is reported to be hyper-activated (hyperautoacetylated) in oral cancer. Probably, the activated p300 causes hyper-crotonylation, and other protein factors cause the functional translation of this modification. The authors need to clarify this with a suitable experiment.

We thank the reviewer for this insightful comment regarding the functional relationship between YEATS2 and p300 in the context of H3K27cr, especially considering reports of p300 hyper-activation in oral cancer. We agree that a precise clarification of p300's role and its cooperativity with YEATS2 is crucial to fully understand the functional translation of this modification.

As we have shown through global RNA-seq and ChIP-seq analyses, YEATS2 broadly affects gene expression by regulating histone crotonylation levels at gene promoters. We also recognize that the histone writer p300 is a promiscuous acyltransferase, known to add various non-acetyl marks, including crotonylation[4]. Our initial data, showing decreased p300 occupancy on the *SPARC* promoter upon YEATS2 downregulation (Figure 5F), suggested a strong dependency of p300 on YEATS2 for its recruitment. To fully delineate the molecular mechanism of this cooperativity and clarify how YEATS2 influences p300-mediated histone crotonylation and its functional outcomes, we have performed the following series of experiments, which have been integrated into the revised manuscript:

(a) Validation of p300's role in SPARC expression: We performed p300 knockdown in BICR10 cells, followed by immunoblotting to assess SPARC protein levels. As expected, a significant decrease in SPARC protein levels was observed upon p300 knockdown (Figure 5G). This confirms p300's direct involvement in SPARC gene expression.

(b) Direct interaction between YEATS2 and p300: To investigate a potential physical association, we performed co-immunoprecipitation assays to check for an interaction between endogenous YEATS2 and p300. Our results clearly demonstrate the presence of YEATS2 in the p300-immunoprecipitate sample, indicating that YEATS2 and p300 physically interact and likely function together as a complex to drive the expression of target genes like SPARC (Figure 5H). This direct interaction provides the mechanistic basis for how YEATS2 influences p300 occupancy.

(c) Impact on transcriptional activity (Pol II recruitment): As suggested, we performed RNA Polymerase II (Pol II) ChIP-qPCR on the *SPARC* promoter in YEATS2 knockdown cells. We observed a significant decrease in Pol II occupancy on the *SPARC* promoter after YEATS2 knockdown in BICR10 cells (Figure 6C). This confirms that YEATS2 silencing leads to reduced transcriptional initiation/elongation at this promoter.

(d) p300's direct role in H3K27cr on *SPARC* promoter: To confirm p300's specific role in crotonylation at this locus, we performed H3K27cr ChIP-qPCR after p300 knockdown. As anticipated, a significant decrease in H3K27cr enrichment was observed on the *SPARC* promoter upon p300 knockdown (Figure 6J), directly demonstrating p300's crotonyltransferase activity at this site.

(e) Rescue of p300 occupancy and H3K27cr by YEATS2 overexpression in SP1-deficient cells: To further establish the YEATS2-p300 axis, we performed SP1 knockdown (which reduces YEATS2 expression) followed by ectopic YEATS2 overexpression, and then assessed p300 occupancy and H3K27cr levels on the *SPARC* promoter. While SP1 knockdown led to a decrease in both p300 and H3K27cr enrichment, we observed a significant rescue of both p300 occupancy and H3K27cr enrichment upon YEATS2 overexpression in the sh*SP1* cells (Figure 6K and L). This provides strong evidence that YEATS2 acts downstream of SP1 to regulate p300 recruitment and H3K27cr levels.

Collectively, these comprehensive new results clearly establish that YEATS2 directly interacts with and assists in the recruitment of p300 to the *SPARC* promoter. This recruitment is crucial for p300's localized crotonyltransferase activity, leading to increased H3K27cr marks and subsequent activation of *SPARC* transcription. This clarifies the previously observed 'paradox' and defines a novel cooperative mechanism between a histone reader (YEATS2) and a writer (p300) in regulating histone crotonylation and gene expression.

(5) I do not entirely agree with using GAPDH as a control in the western blot experiment since GAPDH has been reported to be overexpressed in oral cancer.

We would like to clarify that GAPDH was not used as a loading control for protein expression comparisons between normal and tumor samples. GAPDH was used as a loading control only in experiments using head and neck cancer cell lines where shRNA-mediated knockdown or overexpression was employed. These manipulations specifically target the genes of interest and are not expected to alter GAPDH expression, making it a suitable loading control in these instances.

(6) The expression of EMT markers has been checked in shControl and shYEATS2 transfected cell lines (Figure 2A). However, their expression should first be checked directly in the patients' normal vs. tumor samples.

We thank the reviewer for the suggestion. We have now checked the expression of EMT marker Twist1 alongside YEATS2 expression in normal vs. tumor tissue samples using IHC (Figure 4E).

(7) In Figure 3G, knockdown of SP1 led to the reduced expression of YEATS2 controlled gene Twist1. Ectopic expression of YEATS2 was able to rescue Twist1 partially. In order to establish that SP1 directly regulates YEATS2, SP1 should also be re-introduced upon the knockdown background along with YEATS2 for complete rescue of Twist1 expression.

To address the reviewer’s concern regarding the partial rescue of Twist1 in SP1 depleted-YEATS2 overexpressed cells, we performed the experiment as suggested by the reviewer. We overexpressed both SP1 and YEATS2 in SP1-depleted cells and found that Twist1 depletion was almost completely rescued (Author response image 2).

Author response image 2

**Author response image 2.** 

Immunoblot depicting the decreased Twist1 levels on SP1 knockdown and its subsequent rescue of expression upon YEATS2 and SP1 overexpression in BICR10 (endogenous YEATS2 band indicated by *).

(8) In Figure 7G, the expression of EMT genes should also be checked upon rescue of SPARC expression.

We thank the reviewer for the suggestion. We have examined the expression of EMT marker Twist1 on YEATS2/ GCDH rescue. On overexpressing both YEATS2 and GCDH in sh*SP1* cells we found that the depleted expression of Twist1 was rescued (Author response image 3).

Author response image 3

**Author response image 3.** 

Immunoblot depicting the decreased Twist1 levels on SP1 knockdown and its subsequent rescue of expression upon dual overexpression of YEATS2 and GCDH in BICR10 (* indicates GFP-tagged YEATS2 probed using GFP antibody).

**Reviewer #1 (Recommendations for the authors):**

While the study offers insights into the specific role of this axis in regulating epithelial-tomesenchymal transition (EMT) in HNC, its broader mechanistic novelty is limited by prior discoveries in other cancer types (https://doi.org/10.1038/s41586-023-06061-0). The manuscript would benefit from the inclusion of metastasis data, the role of key metabolic enzymes like ECHS1, the molecular mechanisms governing p300 and YEATS2 interactions, additional IHC data, negative control data in ChIP, and an explanation of discrepancies in certain figures.

We thank the reviewer for their constructive suggestions. We have made extensive revisions to our manuscript to substantiate our findings. We have looked into the expression of ECHS1/ GCDH in HNC tumor tissues using IHC, performed extensive experiments to validate the role of p300 in YEATS2-mediated histone crotonylation, and provided additional data supporting our findings wherever required. The revised figures have been provided in the updated version of the manuscript and also in the Authors’ response.

Minor Comments:

(1) The study begins with a few EMT markers, such as Vimentin, Twist, and N-Cadherin to validate the role of YEATS2 in promoting EMT. Including a broader panel of EMT markers would strengthen the conclusions about the effects of YEATS2 on EMT and invasion. Additionally, the rationale for selecting these EMT markers is not fully elaborated. Why were other well-known EMT players not included in the analysis?

On performing RNA-seq with shControl and sh_YEATS2_ samples, we discovered that *TWIST1* was showing decrease in expression on YEATS2 downregulation. So Twist1 was investigated as a potential target of YEATS2 in HNC cells. N-Cadherin was chosen because it is known to get upregulated directly by Twist1[8]. Further, Vimentin was chosen as it a well-known marker for mesenchymal phenotype and is frequently used to indicate EMT in cancer cells[9].

Author response image 4

**Author response image 4.** 

IGV plot showing the decrease in Twist1 expression in shControl vs. shYEATS2 RNA-seq data.

Other than the EMT-markers used in our study, the following markers were amongst those that showed significant change in gene expression on YEATS2 downregulation.

Author response table 1

**Author response table 1.** 

HUGO Gene Symbol	Common Name	Marker Type	Status in shControl vs. shYEATS2 RNA-seq
COL4A1	Collagen IV alpha 1	Epithelial	Downregulated
FN1	Fibronectin	Mesenchymal	Downregulated
S100A4	S100A4	Mesenchymal	Downregulated
CTNNB1	Beta-catenin	Dual	Downregulated
CDH11	Cadherin-11	Dual	Downregulated
MMP2	MMP2	Mesenchymal	Downregulated
TWIST1	Twist1	Mesenchymal	Downregulated
CDH1	E-cadherin	Epithelial	Upregulated
GSK3B	GSK3B	Dual	Upregulated
SDC1	Syndecan-1	Epithelial	Upregulated
CLDN1	Claudin-1	Epithelial	Upregulated
			

List of EMT-related genes that showed significant change in expression on YEATS2 knockdown in RNA-seq analysis.

As depicted in the table above, majority of the genes that showed downregulation on YEATS2 knockdown were mesenchymal markers, while epithelial-specific genes such as Ecadherin and Claudin-1 showed upregulation. This data signifies the essential role of YEATS2 in driving EMT in head and neck cancer.

(2) The authors use Ponceau staining, but the rationale behind this choice is unclear. Ponceau is typically used for transfer validation. For the same patient, western blot loading controls like Actin/GAPDH should be shown. Also, at various places throughout the manuscript, Ponceau staining has been used. These should also be replaced with Actin/GAPDH blots.

Ponceau S staining is frequently used as alternative for housekeeping genes like GAPDH as control for protein loading[10]. However, to address this issue, we have repeated the western and used H3 as a loading control as nuclear protein lysates from patient samples were used to check YEATS2 and H3K27cr levels.

For experiments (In Figures 5E, 6F, 6I, and 7H) where we assessed SPARC levels in conditioned media obtained from BICR10 cells (secretory fraction), Ponceau S staining was deliberately used as the loading control. In such extracellular protein analyses, traditional intracellular housekeeping genes (like Actin or GAPDH) are not applicable. Ponceau S has been used as a control for showing SPARC expression in secretory fraction of mammalian cell lines in previous studies as well11.

(3) The manuscript briefly mentions that p300 was identified as the only protein with increased expression in tumours compared to normal tissue in the TCGA dataset. What other writers were checked for? Did the authors check for their levels in HNC patients?

We thank the reviewer for this observation. As stated by previous studies [12,13], p300 and GCN5 are the histone writers that can act as crotonyltransferases at the H3K27 position. Although the crotonyltransferase activity of GCN5 has been demonstrated in yeast, it has not been confirmed in human. Whereas the histone crotonyltransferase activity of p300 has been validated in human cells using in vitro HCT assays[4,14]. Therefore, we chose to focus on p300 for further validation of its role in YEATS2mediated regulation of histone crotonylation. We did not check the levels of p300 in HNC patient tissues. However, p300 showed higher expression in tumor as compared to normal in publicly available HNC TCGA RNA-seq data (Figure 5—figure supplement 1G).

We acknowledge that the original statement in the manuscript, 'For this we looked at expression of the known writers of H3K27Cr mark in TCGA dataset, and discovered that p300 was the only protein that had increased expression in tumor vs. normal HNC dataset…', was indeed slightly misleading. Our intention was to convey that p300 is considered the major and most validated histone crotonyltransferase capable of influencing crotonylation at the H3K27 position in humans, and that its expression was notably increased in the HNC TCGA tumor dataset. We have now reframed this sentence in the revised manuscript to accurately reflect our findings and focus, as follows:

'For this, we checked the expression of p300, a known writer of H3K27cr mark in humans, in the TCGA dataset. We found that p300 had increased expression in tumor vs. normal HNC dataset…'

This revised wording more accurately reflects our specific focus on p300's established role and its observed upregulation in HNC.

(4) Figure 6E, blot should be replaced. The results aren't clearly visible.

We thank the reviewer for this observation. We have repeated the western blot and the Figure 6E (Figure 6F in the revised version of manuscript) has now been replaced with a cleaner blot.

(5) Reference 9 and 19 are the same. Please rectify.

We apologize for this inadvertent error. We have rectified this error in the updated version of the manuscript.

References

(1) Brabletz, T.; Kalluri, R.; Nieto, M. A.; Weinberg, R. A. EMT in Cancer. Nat Rev Cancer 2018, 18(2), 128–134. https://doi.org/10.1038/nrc.2017.118.

(2) Pisani, P.; Airoldi, M.; Allais, A.; Aluffi Valletti, P.; Battista, M.; Benazzo, M.; Briatore, R.; Cacciola, S.; Cocuzza, S.; Colombo, A.; Conti, B.; Costanzo, A.; Della Vecchia, L.; Denaro, N.; Fantozzi, C.; Galizia, D.; Garzaro, M.; Genta, I.; Iasi, G. A.; Krengli, M.; Landolfo, V.; Lanza, G. V.; Magnano, M.; Mancuso, M.; Maroldi, R.; Masini, L.; Merlano, M. C.; Piemonte, M.; Pisani, S.; Prina-Mello, A.; Prioglio, L.; Rugiu, M. G.; Scasso, F.; Serra, A.; Valente, G.; Zannetti, M.; Zigliani, A. Metastatic Disease in Head & Neck Oncology. Acta Otorhinolaryngol Ital 2020, 40 (SUPPL. 1), S1–S86. https://doi.org/10.14639/0392-100X-suppl.1-40-2020.

(3) Lin, J.; Zhang, P.; Liu, W.; Liu, G.; Zhang, J.; Yan, M.; Duan, Y.; Yang, N. A Positive Feedback Loop between ZEB2 and ACSL4 Regulates Lipid Metabolism to Promote Breast Cancer Metastasis. Elife 2023, 12, RP87510. https://doi.org/10.7554/eLife.87510.

(4) Liu, X.; Wei, W.; Liu, Y.; Yang, X.; Wu, J.; Zhang, Y.; Zhang, Q.; Shi, T.; Du, J. X.; Zhao, Y.; Lei, M.; Zhou, J.-Q.; Li, J.; Wong, J. MOF as an Evolutionarily Conserved Histone Crotonyltransferase and Transcriptional Activation by Histone Acetyltransferase-Deficient and Crotonyltransferase-Competent CBP/P300. Cell Discov 2017, 3 (1), 17016. https://doi.org/10.1038/celldisc.2017.16.

(5) Jiang, G.; Li, C.; Lu, M.; Lu, K.; Li, H. Protein Lysine Crotonylation: Past, Present, Perspective. Cell Death Dis 2021, 12 (7), 703. https://doi.org/10.1038/s41419-021-03987-z.

(6) Yuan, H.; Wu, X.; Wu, Q.; Chatoff, A.; Megill, E.; Gao, J.; Huang, T.; Duan, T.; Yang, K.; Jin, C.; Yuan, F.; Wang, S.; Zhao, L.; Zinn, P. O.; Abdullah, K. G.; Zhao, Y.; Snyder, N. W.; Rich, J. N. Lysine Catabolism Reprograms Tumour Immunity through Histone Crotonylation. Nature 2023, 617 (7962), 818–826. https://doi.org/10.1038/s41586-023-06061-0.

(7) Zhao, D.; Guan, H.; Zhao, S.; Mi, W.; Wen, H.; Li, Y.; Zhao, Y.; Allis, C. D.; Shi, X.; Li, H. YEATS2 Is a Selective Histone Crotonylation Reader. Cell Res 2016, 26 (5), 629–632. https://doi.org/10.1038/cr.2016.49.

(8) Alexander, N. R.; Tran, N. L.; Rekapally, H.; Summers, C. E.; Glackin, C.; Heimark, R. L. NCadherin Gene Expression in Prostate Carcinoma Is Modulated by Integrin-Dependent Nuclear Translocation of Twist1. Cancer Res 2006, 66 (7), 3365–3369.

https://doi.org/10.1158/0008-5472.CAN-05-3401.

(9) Satelli, A.; Li, S. Vimentin in Cancer and Its Potential as a Molecular Target for Cancer Therapy. Cellular and Molecular Life Sciences 2011, 68 (18), 3033–3046. https://doi.org/10.1007/s00018-011-0735-1.

(10) Romero-Calvo, I.; Ocón, B.; Martínez-Moya, P.; Suárez, M. D.; Zarzuelo, A.; Martínez-Augustin, O.; de Medina, F. S. Reversible Ponceau Staining as a Loading Control Alternative to Actin in Western Blots. Anal Biochem 2010, 401 (2), 318–320. https://doi.org/10.1016/j.ab.2010.02.036.

(11) Ling, H.; Li, Y.; Peng, C.; Yang, S.; Seto, E. HDAC10 Inhibition Represses Melanoma Cell Growth and BRAF Inhibitor Resistance via Upregulating SPARC Expression. NAR Cancer 2024, 6 (2), zcae018. https://doi.org/10.1093/narcan/zcae018.

(12) Gao, D.; Li, C.; Liu, S.-Y.; Xu, T.-T.; Lin, X.-T.; Tan, Y.-P.; Gao, F.-M.; Yi, L.-T.; Zhang, J. V; Ma, J.Y.; Meng, T.-G.; Yeung, W. S. B.; Liu, K.; Ou, X.-H.; Su, R.-B.; Sun, Q.-Y. P300 Regulates Histone Crotonylation and Preimplantation Embryo Development. Nat Commun 2024, 15 (1), 6418. https://doi.org/10.1038/s41467-024-50731-0.

(13) Li, K.; Wang, Z. Histone Crotonylation-Centric Gene Regulation. Epigenetics Chromatin 2021, 14 (1), 10. https://doi.org/10.1186/s13072-021-00385-9.

(14) Sabari, B. R.; Tang, Z.; Huang, H.; Yong-Gonzalez, V.; Molina, H.; Kong, H. E.; Dai, L.; Shimada, M.; Cross, J. R.; Zhao, Y.; Roeder, R. G.; Allis, C. D. Intracellular Crotonyl-CoA Stimulates Transcription through P300-Catalyzed Histone Crotonylation. Mol Cell 2015, 58 (2), 203–215. https://doi.org/10.1016/j.molcel.2015.02.029.
